# Mitochondrial *atp1* mRNA knockdown by a custom-designed pentatricopeptide repeat protein alters ATP synthase

**DOI:** 10.1093/plphys/kiae008

**Published:** 2024-01-11

**Authors:** Fei Yang, Lilian Vincis Pereira Sanglard, Chun-Pong Lee, Elke Ströher, Swati Singh, Glenda Guec Khim Oh, A Harvey Millar, Ian Small, Catherine Colas des Francs-Small

**Affiliations:** Australian Research Council Centre of Excellence in Plant Energy Biology, School of Molecular Sciences, The University of Western Australia, Crawley, WA 6009, Australia; State Key Laboratory for Conservation and Utilization of Bio-Resources in Yunnan, Yunnan Agricultural University, Kunming 650201, P. R. China; Australian Research Council Centre of Excellence in Plant Energy Biology, School of Molecular Sciences, The University of Western Australia, Crawley, WA 6009, Australia; Australian Research Council Centre of Excellence in Plant Energy Biology, School of Molecular Sciences, The University of Western Australia, Crawley, WA 6009, Australia; Australian Research Council Centre of Excellence in Plant Energy Biology, School of Molecular Sciences, The University of Western Australia, Crawley, WA 6009, Australia; Australian Research Council Centre of Excellence in Plant Energy Biology, School of Molecular Sciences, The University of Western Australia, Crawley, WA 6009, Australia; Australian Research Council Centre of Excellence in Plant Energy Biology, School of Molecular Sciences, The University of Western Australia, Crawley, WA 6009, Australia; Australian Research Council Centre of Excellence in Plant Energy Biology, School of Molecular Sciences, The University of Western Australia, Crawley, WA 6009, Australia; Australian Research Council Centre of Excellence in Plant Energy Biology, School of Molecular Sciences, The University of Western Australia, Crawley, WA 6009, Australia; Australian Research Council Centre of Excellence in Plant Energy Biology, School of Molecular Sciences, The University of Western Australia, Crawley, WA 6009, Australia

## Abstract

Spontaneous mutations are rare in mitochondria and the lack of mitochondrial transformation methods has hindered genetic analyses. We show that a custom-designed RNA-binding pentatricopeptide repeat (PPR) protein binds and specifically induces cleavage of ATP synthase subunit1 (*atp1*) mRNA in mitochondria, significantly decreasing the abundance of the Atp1 protein and the assembled F_1_F_o_ ATP synthase in *Arabidopsis* (*Arabidopsis thaliana*). The transformed plants are characterized by delayed vegetative growth and reduced fertility. Five-fold depletion of Atp1 level was accompanied by a decrease in abundance of other ATP synthase subunits and lowered ATP synthesis rate of isolated mitochondria, but no change to mitochondrial electron transport chain complexes, adenylates, or energy charge in planta. Transcripts for amino acid transport and a variety of stress response processes were differentially expressed in lines containing the PPR protein, indicating changes to achieve cellular homeostasis when ATP synthase was highly depleted. Leaves of ATP synthase-depleted lines showed higher respiratory rates and elevated steady-state levels of numerous amino acids, most notably of the serine family. The results show the value of using custom-designed PPR proteins to influence the expression of specific mitochondrial transcripts to carry out reverse genetic studies on mitochondrial gene functions and the consequences of ATP synthase depletion on cellular functions in *Arabidopsis*.

## Teaser

Knockdown of mitochondrial *atp1* mRNA by a custom-designed pentatricopeptide repeat protein alters F_1_F_o_ ATP synthase, plant growth, amino acid metabolism, and ATP synthesis in *Arabidopsis*.

## Introduction

Plant mitochondria are semiautonomous organelles that produce ATP, the universal energy currency in the cell, through oxidative phosphorylation, whose final step is catalyzed by the ATP synthase. Due to their endosymbiotic origins (Andersson et al. 2003), they contain their own genome, retaining about 65 functional genes. The scarcity of mitochondrial mutations and the lack of reliable methods to transform mitochondria or knockdown expression of mitochondrial genes (Colas des Francs-Small et al. 2018; Kazama et al. 2019; Niazi et al. 2019) have made genetic analyses difficult (Kubo and Newton 2008).

Although rare, spontaneous recombination of the mitochondrial genome can generate chimeric ORFs encoding proteins that may cause pollen sterility (Chase 2007; Mower et al. 2012). This phenomenon, known as cytoplasmic male sterility (CMS), is an important agronomic trait that has been widely used for plant hybrid seed production (Chen et al. 2017) and can be suppressed by nuclear restorer of fertility (*Rf*) genes (Hu et al. 2012; Huang et al. 2015). However, very few CMS genes have been functionally validated because of the lack of mitochondrial transformation strategies (Kazama et al. 2019), but mitochondrial ATP synthase subunit genes *atp1*, *atp4*, *atp6*, *atp8*, and *atp9* have often been found in CMS-associated loci (Hanson and Bentolila 2004; Chen et al. 2017).

Within the large family of ATPases (Stewart et al. 2014), F_1_F_o_-ATP synthases in the inner mitochondrial membrane (Complex V) produce ATP from ADP and inorganic phosphate (Pi) by rotary catalysis, an essential process common to all forms of life (Kuhlbrandt 2019). The complex consists of more than 17 different subunits (Supplementary Table S1) (Senkler et al. 2017) assembled into a soluble F_1_ sector and a membrane-embedded F_o_ sector, which are joined together by central and peripheral stalks (Artika 2019). Proton translocation through the intermembrane space into the matrix drives the rotation of the F_o_ domain and the attached central stalk (Walker 2013), and the conformational changes of the α (Atp1) and β (Atp2) subunits in F_1_ subsequently catalyze the synthesis of ATP (Srivastava et al. 2018). Although their bacterial and plastid counterparts are monomeric (Daum et al. 2010; Kuhlbrandt 2019), mitochondrial ATP synthase complexes (∼600 kD) arrange in rows of dimers, generating the characteristic curvature of the inner mitochondrial membrane known as cristae (Dudkina et al. 2005; Hahn et al. 2016; Gu et al. 2019), thus increasing the membrane surface and the density of respiratory complexes on a mitochondrial volume basis. ATP synthase dimers occupy the tips of the cristae while Complex I and Complex I to III supercomplexes are limited to the flat sections of the mitochondrial inner membrane (Davies et al. 2011). Tetrameric structures have been reported in mitochondria from some mammals (Gu et al. 2019) and free-living ciliates (Flygaard et al. 2020) but not in plants.

To study energy metabolism related to CMS, several laboratories have attempted to knockdown nucleus-encoded subunits of ATP synthase. When induced during germination, antisense RNA-mediated depletion of oligomycin sensitivity-conferring protein (OSCP/ATP5) and γ (ATP3) subunits leads to seedling lethality, stressing the essential role of Complex V. Lower levels of depletion resulted in altered leaf morphology, redox status, metabolism, and gene expression (Robison et al. 2009). Depletion of the δ subunit by RNAi caused growth retardation, male sterility, female defects, decreased ATP synthase amounts, accumulation of reactive oxygen species, and important metabolic changes (Geisler et al. 2012). Similar changes, as well as increased plant heat sensitivity, were observed in ATPd RNAi lines (Liu et al. 2021). Loss of ATP2 in Chlamydomonas (*Chlamydomonas reinhardtii*) altered mitochondrial and chloroplast ultrastructure and function (Lapaille et al. 2010). Mutations in MALE GAMETOPHYTE DEFECTIVE 1 (*Mgp1*), the gene encoding the F_A_d subunit of the mitochondrial ATP synthase, lowered pollen viability (Li et al. 2010). Further studies have described the effects of expressing either an unedited copy of *atp9* (Busi et al. 2011) or fragments of the *atp4* transcript (Shaya et al. 2012), while others focused on mutants affected in the expression of mitochondrial ATP synthase subunit genes, such as organelle transcript processing 87 (*otp87*) (Hammani et al. 2011; Colas des Francs-Small and Small 2013).

In recent years, in the absence of a reliable mitochondrial transformation method, several indirect approaches aiming to alter mitochondrial gene expression have been attempted. Targeted knockdown of mitochondrial gene expression was achieved via tRNA-like ribozymes (Val et al. 2011; Sultan et al. 2016; Niazi et al. 2019). In another approach, transcription activator-like effector nucleases (TALENs) targeted to mitochondria were used to knock out the CMS-associated genes *orf79* in rice (*Oryza sativa*) and *orf125* in rapeseed (*Brassica napus* subsp. *napus*) (Kazama et al. 2019) or to generate targeted mutations in mitochondrial genomes (Kazama et al. 2019; Forner et al. 2022).

We developed a different approach for reverse genetics in plant mitochondria, which successfully induced cleavage of NADH dehydrogenase 6 (*nad6*) transcripts by a custom-designed pentatricopeptide repeat (PPR) protein (Colas des Francs-Small et al. 2018). PPR proteins are organelle RNA-binding proteins that have more than 400 members in most species of land plants (Barkan and Small 2014). The protein family is divided into subgroups according to the length and disposition of their repeated motifs (Cheng et al. 2016). They bind organelle transcripts in a sequence-specific manner (Yin et al. 2013; Yan et al. 2019) and affect the editing, processing, splicing, or translation of the target RNA (Schmitz-Linneweber and Small 2008). Most fertility restorer (*Rf*) genes encode PPR proteins (Kim and Zhang 2018) and form a small clade together with restorer-of-fertility-like (RFL) proteins (Fujii et al. 2011; Melonek et al. 2016; Anisimova et al. 2019; Melonek et al. 2019). RF proteins interact with and block the translation of CMS transcripts by inducing cleavage, preventing ribosome translocation (Dahan and Mireau 2013; Gaborieau et al. 2016; Wang et al. 2021). Among 26 RFL proteins in *Arabidopsis* (*Arabidopsis thaliana*), several functionally characterized members are also known to induce cleavage of mitochondrial transcripts (Jonietz et al. 2010; Holzle et al. 2011; Arnal et al. 2014; Stoll et al. 2017).

The PPR code describing how 2 amino acids in each PPR protein repeat recognize each base of its target RNA provides a strategy to design PPR proteins for plant mitochondrial RNA manipulation (Barkan et al. 2012; Yan et al. 2019). We previously redesigned the RFL protein RPF2 (also known as RFL6) to bind within the *nad6* transcript coding sequence, promoting its cleavage (Colas des Francs-Small et al. 2018). This led to undetectable amounts of Nad6 subunit and consequently undetectable amounts of assembled Complex I, the first complex of the respiratory chain. The high specificity of PPR proteins for their targets allowed us to effectively and precisely knockdown *nad6*, as shown by the few off-target effects. If generalizable, this method would be very useful for altering mitochondrial transcript abundance and studying the control of expression of the building blocks of multisubunit enzyme complexes and their assembly process.

In this work, we show that a modified RFL protein (RPF2-*atp1)* designed to bind the coding sequence of mitochondrial *atp1* can effectively knockdown *atp1* transcripts in vivo, causing delayed growth and reduced fertility in *Arabidopsis*. How these plants can develop and reproduce under these deleterious conditions is explored.

## Results

### RPF2-*atp1* protein design and primary transformant screening in T1 generation

**Figure 1.**
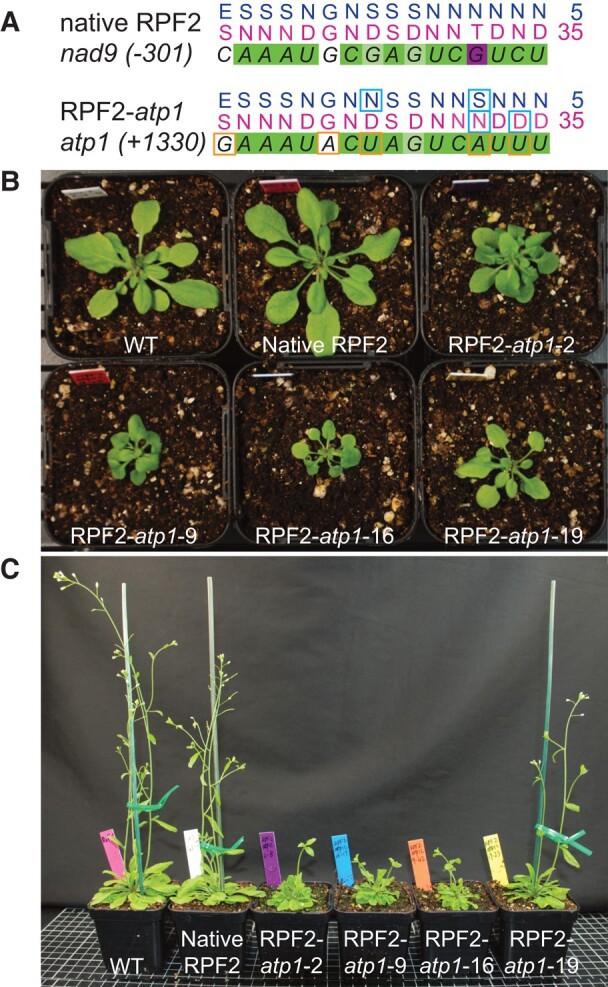
Targeting the *atp1* transcript with a modified RPF2 protein leads to delayed plant growth and curled leaf phenotype. **A)** RNA targets for RPF2 (in *nad9* 5′-UTR) and RPF2-*atp1* (in *atp1* coding sequence) and respective binding predictions. Dark green squares represent a perfect match, light green a partial match, white a neutral match, and magenta a mismatch according to the PPR code. The differences between RNA targets are highlighted by orange squares and the modifications in the protein by cyan squares. **B)** and **C**) show the phenotypes of 4 lines (T2 generation) transformed with the RPF2-*atp1* and native RPF2 as compared with WT. **B**) Four-week-old rosettes. **C**) Six-wk-old plants grown under 18-h photoperiod.

The native RPF2 protein (At1g62670) is composed of 16 PPR motifs (Fig. 1A). It participates in mitochondrial mRNA maturation by binding the 5′-UTRs of *cox3* and *nad9* and inducing cleavage (Jonietz et al. 2010). Using the online program EMBOSS: fuzznuc, we found a sequence at position +1330 to 1346 in the coding sequence of *atp1* (coordinates 67292 to 67277 on the Col-0 mitochondrial genome BK010421, Sloan et al. 2018) with only 5 differences to the predicted RPF2-binding site in the *nad9* transcript (Fig. 1A). The coding sequence of RPF2 was modified to produce a designed protein (RPF2-*atp1*) able to recognize the *atp1* mRNA target sequence (Fig. 1A and Supplementary Fig. S1). *A. thaliana* Col-0 plants were transformed with the synthetic construct via *Agrobacterium tumefaciens* infection. Integration of the construct into the genome was checked in primary transformants (T1) by genomic PCR (Supplementary Fig. S2A). Fourteen out of 32 independent transformants carrying the RPF2-*atp1* construct displayed slow growth and delayed flowering (Fig. 1, B and C). Plants carrying the native RPF2 cDNA and the formate dehydrogenase (FDH) targeting sequence (Colas des Francs-Small et al. 2018) were used as controls. The expression of the RPF2-*atp1* construct in transgenic lines was verified by reverse transcription PCR (RT-PCR) and western blotting (Supplementary Fig. S2, B and C). An RT quantitative PCR (RT-qPCR) experiment performed on 4 independent RPF2-*atp1* lines (RPF2-*atp1-*2, 9, 16, and 19) and on the control plants confirmed that high expression of RPF2-*atp1* reduced *atp1* transcript accumulation (Supplementary Fig. S2B).

### The *atp1* transcript is cleaved in RPF2-*atp1* plants

**Figure 2.**
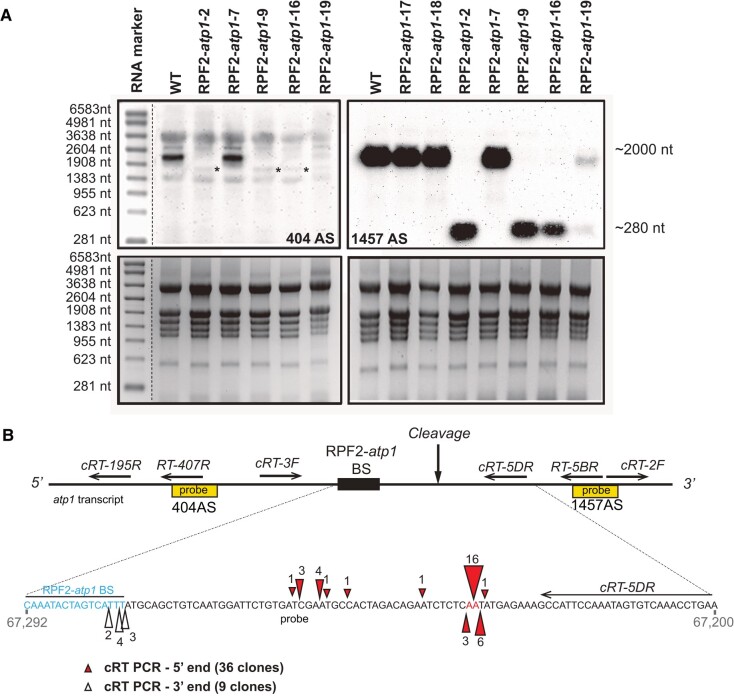
The *atp1* transcript is cleaved in the plants transformed with the RPF2-*atp1* constructs. **A)** Northern blots of leaf RNA isolated from several transformants and WT plants hybridized with *atp1* probes 404AS (top left) and 1457AS (top right), respectively, located upstream and downstream of the RPF2-*atp1* predicted binding site (BS). The bottom panels show the gels stained with ethidium bromide. The molecular weight marker sizes are indicated on the left-hand side. The asterisks on the 404 AS blot show the faint cleavage bands around 1,760 nt. **B)** Location of *atp1* cleavage in the RPF2-*atp1* plants from cRT-PCR results. The top panel indicates the position of the northern probes used in **A)** (yellow boxes) and cRT PCR primers relative to the predicted binding site (not to scale). The coordinates of the enlarged region on the Col-0 mitochondrial genome BK010421 are 67292 to 67200 (reverse strand). The RPF2-*atp1* binding site (67277 to 67292) and the main cleavage sites are highlighted. Red triangles indicate the 5′-ends of the cleaved products and white triangles the 3′-ends of the cleaved products as determined by cRT-PCR. The ends of the 36 clones aligned suggest cleavage between bases 67,224 and 67,225. The figures near the triangles indicate the numbers of clones obtained.

To check for cleavage of the *atp1* transcript in T1 generation without compromising plant viability, northern blotting was carried out on RNA extracted from single rosette leaves of 4-wk-old transformants (Fig. 2A). The *atp1*-specific biotinylated oligonucleotide probe 404 AS (upstream of the predicted binding site) hybridized to a single ∼2,044-nt transcript in wild-type (WT) samples corresponding to the expected size of the mature *atp1* transcript (1,524 nt of coding sequence and around 520 nt of 5′- and 3′-UTRs), but in most RPF2-*atp1* plants, this hybridization signal was very weak and accompanied by an additional faint signal migrating around 1,760 nt (asterisks in Fig. 2A), suggesting that the *atp1* transcript was indeed cleaved in the RPF2-*atp1* plants. With probe 1457AS (hybridizing 3′ of the predicted binding site), a very strong signal corresponding to a potential cleavage product was detected only in the RPF2-*atp1* lines, migrating at ∼280 nt in the RPF2-*atp1*-2, RPF2-*atp1*-9, and RPF2-*atp1*-16 plants (Fig. 2A). In RPF2-*atp1*-19 plants, 2 bands of ∼2,044 and ∼280 nt were detected, indicating that *atp1* transcripts were partially cleaved. Circular RT-PCR (cRT-PCR) experiments were conducted in 2 independent lines (RPF2-*atp1*-9 and RPF2-*atp1*-16), to precisely locate the cleavage site. The cRT-PCR products were cloned and sequenced. Multiple transcript ends could be mapped within a region encompassing 55 nt beginning at or near the end of the predicted RPF2-*atp1* binding site (Fig. 2B).

### RPF2-*atp1* plants have decreased abundance of Complex V and Atp1

**Figure 3.**
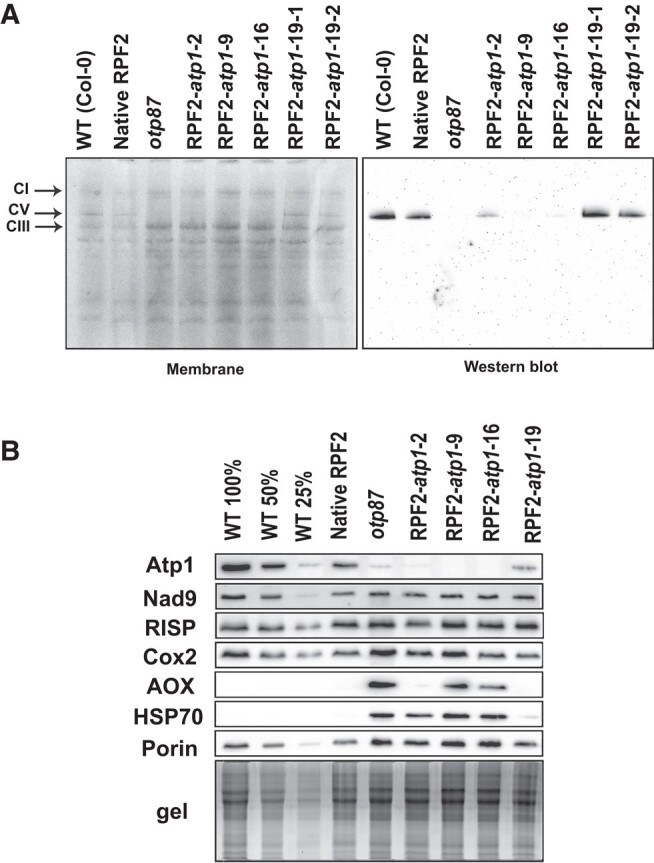
RPF2-*atp1* plants lack Atp1 subunit and assembled respiratory Complex V. **A)** Separation of crude membrane protein complexes by BN–PAGE of 4 RPF2-*atp1* transformants as compared with WT and *otp87* (an *atp1* editing mutant). The left panel shows the stained membrane after transfer and the right panel the western blot probed with an anti-Atp1 antibody. The black arrows show Complexes I (CI), V (CV), and III (CIII). **B)** Western blots of mitochondrial proteins of 4 RPF2-*atp1* transformants as compared with WT, native RPF2, and *otp87* separated by SDS–PAGE.

Blue native PAGE (BN–PAGE) was performed on crude leaf membranes from T2 plants of the transformants to check the integrity of the F_1_F_o_ ATP synthase complex (Fig. 3A). Western blotting of the BN–PAGE gel and probing with an antibody raised against Atp1 revealed assembled Complex V in the mitochondria of WT plants, WT plants transformed with native RPF2 (referred to hereafter as native RPF2 plants) and RPF2-*atp1*-19-1 and 19-2 plants, but reduced amounts in mitochondria from RPF2-*atp1*-2 plants and almost undetectable levels in mitochondria from RPF2-*atp1*-9, RPF2-*atp1*-16, and *otp87* plants (Fig. 3A). Further analysis of respiratory complex subunits by SDS–PAGE and western blotting showed that Nad9 (Complex I), RISP (Complex III), and Cox2 (Complex IV) subunits were unchanged in abundance in RPF2-*atp1* mitochondria, but Atp1 was undetected in mitochondria of RPF2-*atp1*-9 and RPF2-*atp1*-16 plants using this technique (Fig. 3B) and lower in abundance in RPF2-*atp1*-2 and RPF2-*atp1*-19 mitochondria than in WT mitochondria. Because the *atp1* transcript cleavage is partial in the RPF2-*atp1*-19 line (Fig. 2), a small amount of Atp1 was detected, allowing assembly of some Complex V (Fig. 3A). Furthermore, alternative oxidase (AOX) and HSP70 were more abundant in RPF2-*atp1*-9 and 16 plants (Fig. 3B), as observed for other mutants with altered Complex V function (Hammani et al. 2011; Kerbler et al. 2019).

### The modified plants show small rosettes, delayed growth, lower seed production, but increased respiration rates

In T3 generation, the traits of the modified plants were comprehensively investigated. In Week 4, the rosette diameters in RPF2-*atp1*-2, 9, 16, and 19 were only half those of WT and native RPF2 plants. In Week 6, RPF2-*atp1*-2, 9, and 16 still had much smaller rosettes and shorter plant height than control plants; RPF2-*atp1*-19 was similar to WT and native RPF2 plants, in accordance with the *atp1* transcript partial cleavage (Fig. 1B; Supplementary Figs. S3 and S4). Leaf numbers per plant were similar in all genotypes in Week 4 but stopped increasing in the modified plants in Week 6 (Supplementary Fig. S4A). The rosettes of RPF2-*atp1*-2, 9, and 16 developed downwards curled leaves, clearly different from RPF2-*atp1*-19 and control plants (Supplementary Fig. S3D). Bolting and flowering were delayed in the transgenic lines. RPF2-*atp1*-9 plants were most affected, with few very short siliques, resulting in low seed yield per plant (Fig. 1C; Supplementary Figs. S3, B and C, and S4A). The RPF2-*atp1*-2 line showed some instability across generations and was not analyzed further. Root lengths of 10-d-old seedlings grown on vertical plates (Supplementary Figs. S3E and S4B) were significantly shorter in RPF2-*atp1* lines than in control (*P* = 7.5 10^−44^ for RPF2-*atp1*-16; *P* = 1.1 10^−29^ for RPF2-*atp1*-9).

The oxygen consumption rates of leaves from T3 plants (16/8 h, day/night) for 6 wk were measured by a fluorophore-based oxygen sensor. The average oxygen consumption rates of RPF2-*atp1*-9 and RPF2-*atp1*-16 plants were significantly higher than those of WT (*P*-value 0.005 and 0.001, respectively, using Tukey's honestly significant difference (HSD) test) and native RPF2 plants during the 12 h of measuring time while RPF2-*atp1*-19 average respiration rates were close to that of the controls (Supplementary Fig. S5A). The measurements were repeated on leaves grown under short-day conditions (8/16 h, day/night) to reduce leaf size variability across genotypes. Molar O_2_ consumption was calculated for RPF2-*atp1*-9 and RPF2-*atp1-*16 (34 and 47 plants, respectively), the *otp87* mutant (18 plants) (Hammani et al. 2011), and the native RPF2 and WT controls (31 and 28 plants, respectively). The RPF2-*atp1*-9 and RPF2-*atp1*-16 lines displayed 24% higher oxygen consumption rate than the controls, slightly less than *otp87* (29.7%), but significantly higher rates than the controls (*P* < 0.0001 for both lines as compared to WT using Tukey's HSD test) (Supplementary Fig. S5B).

### RNA-seq confirms reproductive development defects in the RPF2-*atp1*-9 plants and highlights their high level of stress

**Figure 4.**
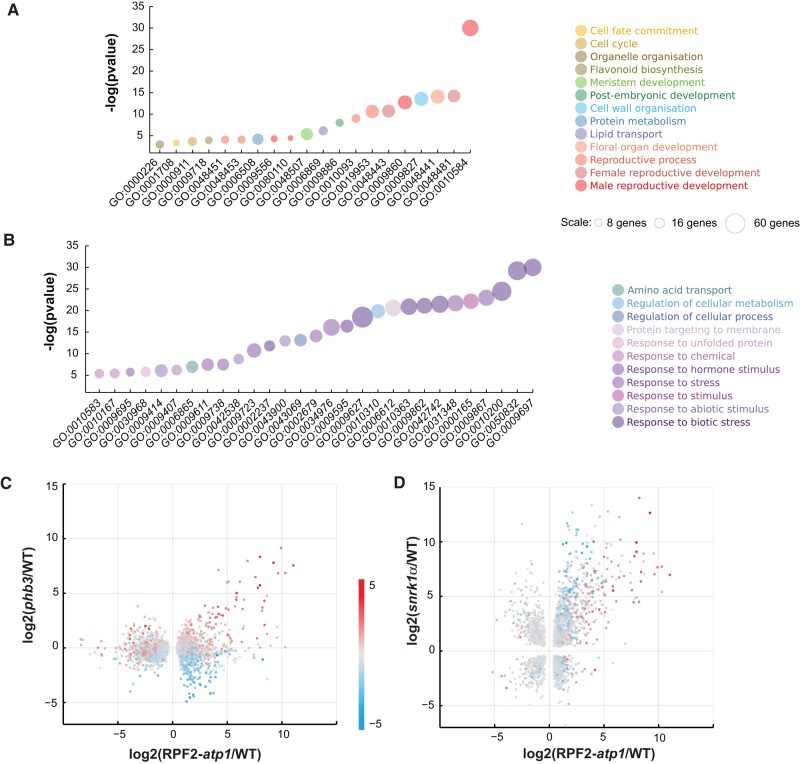
RNA-seq analysis of WT and RPF2-*atp1* plants. **A, B)** GO Biological Process terms most significantly associated with genes more expressed in WT **A)** or in RPF2-*atp1* plants **B)**. Colors indicate groupings of GO terms into broader categories. The area of the markers is proportional to the number of differentially expressed genes in each category (data are available in Supplementary Tables S3 and S4). The *y* axis indicates the degree of statistical significance, higher values being more significant (data are available in Supplementary Tables S3 and S4). **C)** A scatter plot comparing the transcript differences in RPF2-*atp1* and *phb3* mutants (*x* axis is log_2_ fold change between RPF2-*atp1* and WT; *y* axis is log_2_ fold change between *phb3* and WT). Only transcripts that are significantly differentially expressed in RPF2-*atp1* mutants are included. The marker color represents the effect of the ANAC017 transcription factor (calculated as the log_2_ ratio in expression between *phb3* mutants and *phb3 anac017* double mutants). **D)** A scatter plot comparing the transcript differences in RPF2-*atp1* and *snrk1α* mutants (*x* axis is log_2_ fold change between RPF2-*atp1* and WT; *y* axis is log_2_ fold change between *snrk1α* and WT). Only transcripts that are significantly differentially expressed in both mutants are included. The marker color represents the effect of the ANAC017 transcription factor (calculated as the log_2_ ratio in expression between *phb3* mutants and *phb3 anac017* double mutants).

RNA-seq data obtained from 3 biological repeats of the RPF2-*atp1-*9 line (S9 1 to 3) compared to WT (C2 to 4) were analyzed in the T4 generation. Emerging flower buds and young rosette leaves (*Arabidopsis* development stage 5.10) were chosen to ensure high coverage of the mitochondrial transcripts as these tissues are rich in mitochondria. Out of 18,643 transcripts, 5,683 were found to be differentially accumulated using a false discovery rate threshold of 0.1, of which 2,831 were more abundant in RPF2-*atp1*-9 plants and 2,852 were more abundant in WT (Supplementary Table S2). A set of 11 of the gene ontology (GO) terms most significantly overrepresented in the annotations of genes whose expression was lower in RPF2-*atp1*-9 plants are associated with reproductive development (Fig. 4A; Supplementary Table S3). Forty-three out of 45 genes implicated in pollen exine formation, 32 out of 62 genes implicated in pollen tube growth, and 27 out of 48 implicated in stamen development had significantly lower expression levels in the RPF2-*atp1*-9 plants than in WT (Supplementary Table S3; Fig. 4A). GO terms relating to plant growth and development were also highly represented (cell wall organization, postembryonic development, petal development, meristem development, cell cycle, cell fate, and organelle organization) as well as protein metabolism and lipid transport.

Most of the 29 GO terms significantly overrepresented in the terms annotating genes more expressed in RPF2-*atp1*-9 plants (Fig. 4B; Supplementary Table S4) were related to biotic and abiotic stress responses, consistent with the increased levels of AOX and HSP70 observed by western blotting (Fig. 3B). Interestingly, processes such as protein targeting to membrane and response to unfolded protein were also overrepresented as well as amino acid transport.

### RNA-seq shows involvement of the ANAC017 transcription factor and SNRK1 kinase in RPF2-*atp1* line gene expression changes

To assess if transcript increases observed in RPF2-*atp1* lines could be related to the well-documented mitochondrial retrograde signals mediated by the NAC DOMAIN CONTAINING PROTEIN 17 (ANAC017) transcription factor (De Clercq et al. 2013; Ng et al. 2013) and that of prohibitin (PHB3), an inner membrane protein affecting mitochondrial function and stress response (Van Aken et al. 2010), we plotted our RPF2-*atp1* data against RNA-seq data obtained from *phb3* and *phb3 anac017* double mutants (Van Aken et al. 2016). Most of the transcripts strongly induced in RPF2-*atp1* plants are also induced in *phb3* plants and are ANAC017 dependent, but a second group of transcripts induced in RPF2-*atp1* plants are not induced in *phb3* plants and not dependent on ANAC017 (Fig. 4C). In attempting to understand what mediates the induction of these transcripts in the RPF2-*atp1* plants, we compared our RNA-seq data to similar data from a mutant deficient in a SNF1-related protein kinase (SNRK1) involved in metabolic adaptation to low energy supply (Pedrotti et al. 2018). As for the comparison with *phb3* plants, ANAC017-dependent transcripts are induced in both RPF2-*atp1* and *snrk1α* plants, but more so in RPF2-*atp1* plants (Fig. 4D). Interestingly, the ANAC017-independent groups of transcripts induced in RPF2-*atp1* plants but not in *phb3* plants are even more strongly induced in *snrk1α* plants.

### Analysis of mitochondrial transcripts did not reveal any substantial off-target effects of the RPF2-*atp1* protein

**Figure 5.**
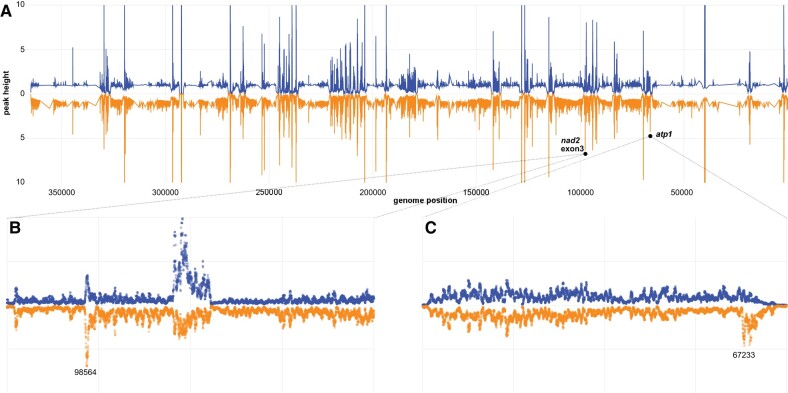
Comparative mapping of RNA-seq 5′-ends confirms cleavage of *atp1* transcripts without significant off-target cleavages. **A)** Relative frequency of RNA 5′-ends mapping to the reverse strand of BK010421 in WT (blue) and RPF2-*atp1* (orange) plants. The data have been smoothed and normalized relative to the local background, are a mean of 3 samples for each genotype, and are shown from 5′ to 3′ (BK010421 coordinates 367808 to 1). The RPF2-*atp1* data are plotted as a mirror image to facilitate comparison to the WT pattern. **B)** Plot of the same data in the region (99000 to 97000) but now showing individual data points for each sample at single-nucleotide resolution. The peaks in the center of the panel indicate the extent of *nad2* exon 3 (BK010421 coordinates 95238 to 94666). The peak in RPF2-*atp1* samples at 98564 (*P* = 0.019) is one of only 2 peaks in the mitochondrial transcriptome to be significantly more prominent in RPF2-*atp1* than in WT. We believe it to indicate the 5′-end of unspliced *nad2* exon3 transcripts. **C)** Plot of the data in the region (69000 to 67000) showing individual data points for each sample at single-nucleotide resolution. This region covers the *atp1* gene (BK010421 coordinates 68621 to 67098). The peak in RPF2-*atp1* samples at 67233 is significantly more prominent in RPF2-*atp1* than in WT (*P* = 0.0099). We believe it to indicate the cleavage site(s) induced by RPF2-*atp1*.

The RNA-seq data were also mined to look for off-target effects due to the potential binding of RPF2-*atp1* on transcripts other than the targeted *atp1*. Confirming the RT-qPCR results, the *atp1* transcript was significantly less abundant in RPF2-*atp1* samples (about 4-fold lower; *P* = 0.004) (Supplementary Fig. S6). The RNA-seq data were also analyzed to specifically search for cleavage events generated by the RPF2-*atp1* protein. Figure 5A shows the relative frequency of RNA-seq 5′-ends across the whole mitochondrial transcriptome for WT and RPF2-*atp1*-9 samples; the profiles for both genotypes are very similar overall. Only 2 prominent peaks are significantly higher in the RPF2-*atp1-9* samples (Fig. 5, B and C), 1 in the *atp1* transcript at position 67,233 (reverse strand), corresponding to the cleavage site induced by the RPF2-*atp1* protein (as determined by cRT-PCR, Fig. 2B), and the other in *nad2* intron 2 (position 98564, reverse strand). This second peak does not represent an RNA cleavage site, but rather the 5′-end of the half-intron (as *nad2* intron 2 is trans-spliced). The increase in abundance of this peak suggested that the splicing efficiency of *nad2* intron 2 might be decreased in RPF2-*atp1-9* plants, so we calculated the splicing efficiencies for all intron-containing mitochondrial transcripts in both genotypes (Supplementary Fig. S7). All introns are significantly less spliced in RPF2-*atp1*-9 plants than in WT with the exception of *nad1.1* (*P* < 0.05 using 1-tailed equal variance *t*-test). Apparent relative editing rates in WT as compared to RPF2-*atp1*-9 were also calculated for 461 mitochondrial editing sites within coding sequences and found to be generally lower in the modified plants (Supplementary Fig. S8).

### Proteomic and metabolomic analyses

**Figure 6.**
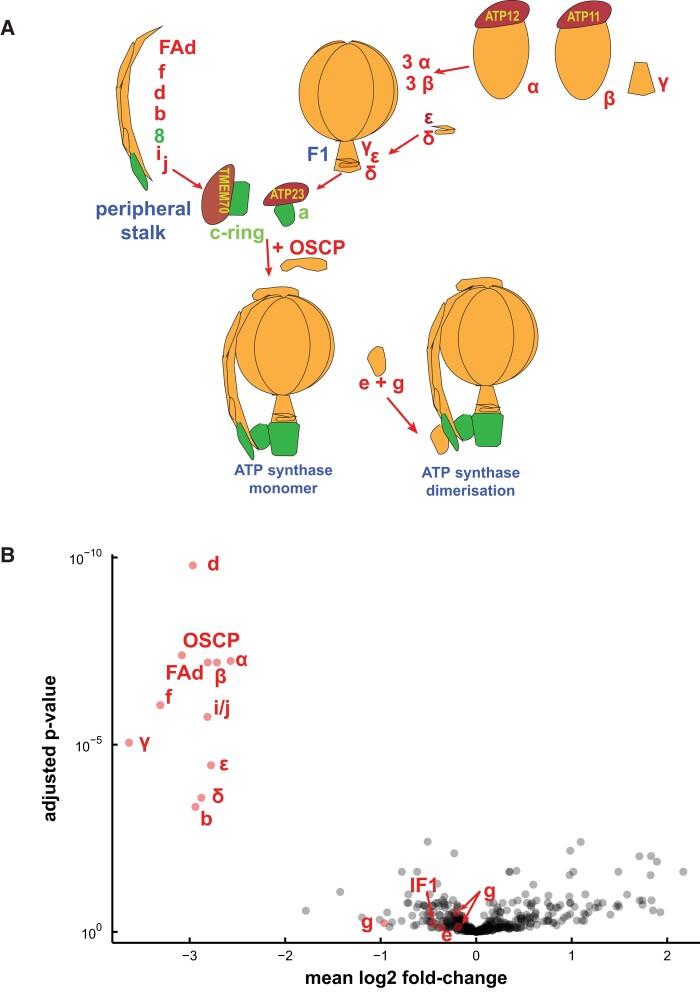
Most subunits of the ATP synthase complex are less abundant in RPF2-*atp1* transformants. **A)** Simplified representation of the ATP synthase assembly pathway. The subunits found in our proteomics study are labeled in red, and the others are labeled in green. **B)** A “volcano” plot of relative protein abundances as estimated by quantitative untargeted MS from samples of WT and RPF2-*atp1* crude mitochondrial pellets. The *x* axis indicates the mean log_2_ fold difference (WT/RPF2-*atp1*); the *y* axis indicates the *P*-value for the hypothesis of equal relative abundance; i.e. points toward the top-left of the plot are significantly less abundant in RPF2-*atp1* samples. The plot shows data for 410 proteins of which 17 (in red) are associated with the ATP synthase complex. The plot is based on MS data from 8 RPF2-*atp1* samples and 8 phenotypically WT samples (4 from WT plants and 4 from plants expressing the native RPF2).

Figure 6A describes the assembly pathway of ATP synthase (adapted from Röhricht et al. 2021) and highlights the other subunits that make up ATP synthase in plant mitochondria. A quantitative untargeted MS approach was used to explore potential changes in mitochondria caused by a decrease in the ATP synthase complex and to check levels of the subunits of the respiratory complexes in RPF2-*atp1-9* and RPF2-*atp1-16* as compared with those in WT and native RPF2. Out of 410 mitochondrial proteins in our analysis, the most impacted in abundance was Atp1 (target of RPF2-*atp1*) and most other components of the ATP synthase complex (Fig. 6B; Supplementary Table S1 and Fig. S9). All components of the F_1_ domain (subunits α, β, γ, δ, and ε) were decreased to 15% to 25% of the WT level, so we can confidently conclude that F_1_ assembly was greatly affected. Most subunits of the peripheral stalk (subunits b, d, OSCP, f, F_A_d, and i/j) followed the same trend, suggesting that the F_o_ domain was not able to assemble properly either. Unfortunately, we could not detect with MS the subunits a (Atp6), 8 (Atp8), and c (Atp9), all highly hydrophobic components of the F_o_ domain.

Unexpectedly, 3 ATP-synthase-associated proteins did not vary: g (ATP21) and e (ATP20), both involved in the dimerization of the F_o_ subcomplex (Zancani et al. 2020; Röhricht et al. 2021) and IF1, an inhibitor factor of ATP synthase activity (Chen et al. 2020). No significant protein changes were detected in subunits of electron transfer chain complexes (Fig. 6B; Supplementary Fig. S9), in accordance with the western blot results (Fig. 3B). Analysis of the RNA-seq data showed induction of transcripts for 2 nonphosphorylating bypasses of the electron transport chain, namely AOX (*AOX1a* and *AOX1d*) and rotenone insensitive NADH dehydrogenase (*NDA1*, *NDB2*, and *NDB4*).

In order to understand how, despite only having 15% to 25% of the mitochondrial ATP synthase found in WT, most RPF2-*atp1* transformants can grow, flower, and set seed, we measured total adenylates in seedling leaves in a preliminary experiment, but in the absence of difference between adenylate levels between “ATP synthase deficient mutant” and WT genotypes (Supplementary Fig. S10A and Table S5-1), they were further measured in a nonphotosynthetic organ. Root tissue was chosen to minimize the fraction of adenylates produced by the chloroplast ATP synthase. These measurements (5 biological repeats) showed that the total root abundance of ATP, ADP, and AMP in the transformants was surprisingly close to those in the control plants with an ATP/ADP ratio of approximately 1.8 and an adenylate charge ((ATP + 0.5 ∗ ADP)/(ATP + ADP + AMP)) of 0.8 (Supplementary Fig. S10B and Tables S5-2 and S6). ATP synthesis rates were directly evaluated in purified substrate-energized mitochondria and found to be 44% and 57% slower in RPF2-*atp1*-16 and RPF2-*atp1*-9, respectively, than in native RPF2 (WT) seedlings (Supplementary Fig. S10C and Table S5-3). This shows that while ATP synthase abundance is substantially lowered, it only has a moderate metabolic control coefficient (∼0.4) on ATP synthesis rate, potentially explaining the ability of RPF2-*atp1-9* and RPF2-*atp1-16* to grow at a slower rate and maintain adenylate pools in vivo.

**Figure 7.**
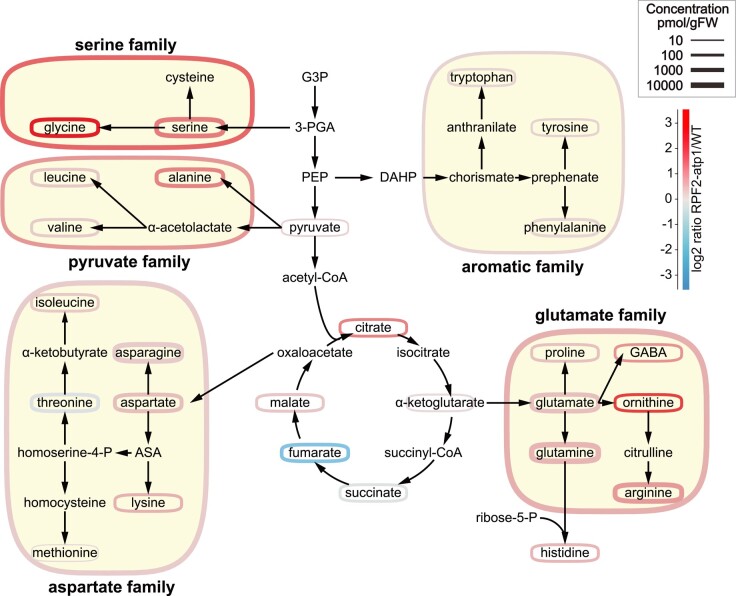
Perturbed metabolite abundances is in the RPF2-*atp1* transformants. Simplified scheme of amino acid synthesis and TCA cycle, adapted from Trovato et al. (2021). The amino acids are grouped by families according to their synthesis pathways. Colors represent the log2 ratio of RPF2-*atp1* to WT concentrations (see scale top right). Outline thickness is proportional to the log of the metabolite or metabolite pool concentration. ASA, aspartate semialdehyde; DAHP, 3-deoxy-Dd-arabinoheptulosonate-7-phosphate; G3P, glyceraldehyde 3-phosphate; 3-PGA, 3-phosphoglycerate; PEP, phosphoenolpyruvate.

To gain further insight into the apparent changes in metabolic processes and changes in amino acid transport processes indicated by transcript profiling (Fig. 4B), absolute organic acid and amino acid levels were measured by LC-MS in seedlings at the end of the night (Supplementary Fig. S11 and Tables S5-4 to S5-6). This revealed substantial changes in metabolite steady-state abundances between the RPF2-*atp1* and WT plants. Citrate and malate accumulated but fumarate was less abundant in the RPF2-*atp1* plants compared to in native RPF2 (WT) controls. All amino acids except threonine accumulated more in RPF2-*atp1* samples than in WT seedlings, suggesting profound changes in amino acid metabolism in the dark in response to a lowered ATP synthase abundance and activity in plants. The most dramatic differences were seen in the concentrations of glycine (Gly), ornithine, serine (Ser), and GABA. Grouping amino acids in families based on their synthesis pathways (Fig. 7) and adding together their absolute abundances (Supplementary Table S7) showed that the aspartate family and aromatic amino acid family increased in abundance the least, pyruvate and glutamate family amino acids doubled in abundance, while Ser family amino acids increased 3- to 4-fold in RPF2-*atp1* plants compared to in WT plants. On a percentage of the total amino acid pool basis, this represented relative homeostasis for glutamate, aromatic, and pyruvate family groups, but a one-third decrease for aspartate family and a 2- to 3-fold increase in Ser family amino acids. This indicates that 20% to 30% of the total amino acid content in the samples are Ser family amino acids in RPF2-*atp1* plants while these amino acids only represent 13% of the total amino acid content in WT plants. Notably, the Gly/Ser ratio increased nearly 4-fold in the RPF2-*atp1* mutants (Supplementary Table S8). The analysis of the set of transcripts for *Arabidopsis* amino acid synthesis and degradation pathways (Hildebrandt 2018) showed a significant induction of transcripts for tryptophan synthesis and degradation enzymes but no consistent change in gene expression for enzymes in other amino acid metabolism families (Supplementary Table S9).

## Discussion

### Knockdown of mitochondrial ATP synthase subunits

We succeeded in knocking down the mitochondrial ATP synthase by PPR-mediated cleavage of the *atp1* transcript, which led to levels of the Atp1 subunit that were 15% to 20% that of the WT (Fig. 6B), compromising the assembly of the ATP synthase complex (Figs. 1 and 3). Although we used the constitutive promoter NOS, we obtained a range of phenotypes, relating to differing levels of expression of the RPF2-*atp1* construct (Supplementary Fig. S2), the most drastic being lethality. The phenotypic consequences of this inactivation in the RPF2-*atp1* plants were consistent with those reported for plants altered in the expression of nuclear subunits of the F_1_F_o_ ATP synthase such as slow growth, downward curled rosette leaf morphology, and male sterility (Robison et al. 2009; Geisler et al. 2012; Shaya et al. 2012; Li et al. 2019). We show that the assembled F_1_F_o_ ATP synthase in these plants was substantially lower in abundance than that in the WT by western blot using a monoclonal antibody raised against Atp1 (single epitope), but the subunits could be accurately quantified as being 15% to 25% for both Atp1 and most subunits of the ATP synthase complex by quantitative MS (Fig. 6). These changes occurred with negligible variation in transcript abundance for any subunits other than Atp1 (Supplementary Fig. S9).

### Independence of ATP synthase abundance from that of electron transport chain complexes

Our proteomic analysis, supported by BN–PAGE and western blots, revealed that only the assembly and function of the ATP synthase complex were affected in the RPF2-*atp1* plants, as subunits of respiratory Complexes I to IV were not significantly affected (Supplementary Fig. S9 and Table S2). This observation was also recently reported for RNAi lines of the d subunit (Liu et al. 2021), suggesting that the biogenesis of the plant ATP synthase is independent from that of the other OXPHOS complexes. In that study, the authors observed a decrease in all ATPase subunits, except e (At3g01130 and At5g15320) and g (At4g29480, At2g19680, and At4g26210) as we did, but also a (Atp6), ε (At1g51650), and ATP23 (At1g51680). In the present study, ε was downregulated in the RPF2-*atp1* plants, like other structural components of the complex but we do not know if subunit a (Atp6) followed the same trend because it was not detected. The e and g subunits are thought to be recruited after an ATP synthase monomer is fully assembled (Röhricht et al. 2021), but as much less complex is formed in RPF2-*atp1*-9 and 16 mitochondria, it is possible that these 2 subunits can persist in the membrane in a residual F_o_ subcomplex. In *Saccharomyces cerevisiae*, the assembly factor ATP23, a metallopeptidase located in the mitochondrial inner membrane, is involved in the processing of the a (Atp6) subunit and its assembly into the F_o_ module (Osman et al. 2007). *Arabidopsis* ATP23 was reported to have lost the ability to cleave the N-terminal extension of Atp6 but still acts as a chaperone to integrate Atp6 into the F_o_ module (Migdal et al. 2017). ATP23 (At3g03420) was not retained in our proteomics analysis after quality filtering, and its transcripts were not accumulated (Supplementary Table S2).

Many CMS-related chimeric ORFs contain parts of genes encoding mitochondrial ATP synthase subunits, while others are adjacent to normal ATP synthase subunit genes (Hanson and Bentolila 2004), supporting the energy deficiency hypothesis to explain the mechanism of CMS (Chen and Liu 2014; Chen et al. 2017). However, how mitochondrial ATP synthase dysfunction leads to abnormal male gamete development remains unclear.

### Links between ATP synthase, mitochondrial dysfunction, and retrograde regulation

Our data provide evidence linking a lowered mitochondrial ATP synthesis rate with general stress responses including induction of *AOX1a* and *AOX1d* gene expression and AOX abundance (Fig. 3B; Supplementary Fig. S9) and a degree of reduced fertility. This suggests that, as was observed in previous studies (Busi et al. 2011; Geisler et al. 2012; Liu et al. 2021), the constitutive reduction of ATP synthase activity by PPR-mediated knockdown of the *atp1* gene can trigger mitochondrial retrograde regulation (Van Aken et al. 2009; Schwarzländer et al. 2012; De Clercq et al. 2013). Our analysis suggests that 2 partly independent transcriptional pathways are activated in the *atp1* mutants (Fig. 4): one is the typical ANAC017-dependent mitochondrial stress pathway, and the other involves transcripts that are on the contrary, repressed by ANAC017 (also repressed by SNRK1α). This study also reports downregulation of male or female gametophyte-specific genes (Fig. 4).

While the exact signaling components linking loss of function of mitochondrial ATP synthase to primary metabolism and development during vegetative and reproductive stages remain to be identified (Meng et al. 2019), the retrograde signaling pathways of energy, hormone, and stress responses activated in the RPF2-*atp1* plants are interesting research targets (Ng et al. 2014; de Souza et al. 2017). The observation that splicing and editing rates of mitochondrial transcripts were reduced in the modified plants is a reminder that editing in particular is sensitive to the physiological state of the plants and thus defects in splicing and editing in mutants are not always indicative of a direct role of the missing protein in these processes.

Plants are very adaptable and use alternative pathways to compensate for deficiencies and maintain homeostasis. Despite having lower amounts of functional mitochondrial ATP synthase and substantially reduced rates of mitochondrial ATP synthesis, the RPF2-*atp1* plants maintain near normal concentrations of ATP in vegetative tissues, as also observed for δ subunit RNAi plants (Geisler et al. 2012). On the other hand, levels of ATP and ADP were reported to be decreased by 35% in the flowers of *atp9*-inactivated *Arabidopsis* plants (Busi et al. 2011), but the ATP/ADP ratio was maintained. The authors suggest that the slow growth of the plants is not due to an energy deficit but due to the long-term metabolic effect of trying to maintain energy homeostasis, and they attribute the male and female fertility defects to the difficulty to maintain that homeostasis in tissues with high energy demand such as reproductive tissues.

### Links between lowering ATP synthase and leaf metabolism in the dark

Knockdown of expression of the gene encoding the delta subunit by RNA interference resulted in partially assembled ATP synthase and defects in pollen development coupled with broad relative changes in the abundance of metabolites in the dark (Geisler et al. 2012). We observed broadly similar organic acid and amino acid changes in our RPF2-*atp1* plants, but by measuring absolute abundance (Supplementary Table S7) rather than relative abundance of amino acids (Geisler et al. 2012), we were also able to combine amino acids and changes in their abundance into amino acid synthesis families to show that the major effect is in the Ser family. The change represents not only a higher abundance but also a marked change in the percentage of the Ser group to amino acids in other families and a 4-fold increase in the Gly/Ser ratio (Fig. 7; Supplementary Table S8). The conversion of Gly to Ser is a major source of NADH in leaf mitochondria in the light that is used by the respiratory chain (Krömer 1995) and as a consequence is a primary source of energy for mitochondrial ATP generation in leaves in the light. Gly accumulation in RPF2-*atp1* lines at the end of the night, both here and in Geisler et al. (2012), is more likely due to energy limitation from respiratory metabolism leading to metabolic adjustments in nonphotorespiratory Ser synthesis pathways associated with glycolysis (Igamberdiev and Kleczkowski 2018) to maintain metabolic homeostasis in vegetative tissues at night. Interestingly, the level of Gly accumulation in RPF2-*atp1*-9 plants was substantially higher than that in RPF2-*atp1*-16 plants (Supplementary Table S7), consistent with the larger decrease in ATP synthase in RPF2-*atp1*-9 than in RPF2-*atp1*-16 plants. The upregulation of *AOX1a*, *AOX1d*, *NDA1*, *NDB2*, and *NDB4* in RPF2-*atp1* plants could help to dissipate high levels of reducing equivalents but may not be sufficient to allow adequate Ser family metabolism in RPF2-*atp1* lines. There was no evidence of changes in the expression of genes for GDC subunits in RPF2-*atp1* plants. While there was a marked induction of the cytosolic *SHM5* (At4g13890), it is a root-specific isoform and its role in Ser hydroxymethyl transfer is uncertain (Nogues et al. 2022) (Supplementary Table S2).

### Methods for targeted knockdown of mitochondrial genes

CMS has been widely used in hybrid seed production, and many CMS-related genes were found by comparing the mitochondrial genomes of sterile and fertile plants (Chen et al. 2017; Kim and Zhang 2018). However, few of the candidate CMS-related genes have been functionally validated because of the lack of mitochondrial transformation strategies (Kazama et al. 2019). In recent years, 3 methods were proposed to target knockdown mitochondrial transcripts or genes via synthetic ribozymes (Val et al. 2011; Sultan et al. 2016; Niazi et al. 2019), designed PPR proteins (Colas des Francs-Small et al. 2018), or mito-TALENs (Kazama et al. 2019). Although proposed earlier (Val et al. 2011), the synthetic ribozyme strategy has not been widely used because of the complex chimeric structure of tRNA-like and custom ribozymes. Nevertheless, the knockdown of MatR by this method allowed a better understanding of this elusive but essential maturase encoded in a mitochondrial intron (Sultan et al. 2016). The mito-TALEN method gave direct evidence that *orf79* in rice and *orf125* in *Brassica* are the causes of CMS (Kazama et al. 2019). The knockdown of *atp1* in mitochondria via the redesigned RPF2 resulted in delayed growth and partial sterility in *Arabidopsis*, due to the lack of α subunit, and therefore of functional ATP synthase, indicating a highly efficient knockdown effect of the target gene by the designed PPR protein. TALEN-based technologies provide a permanent total knockout via DNA alteration (Boettcher and McManus 2015), but designed PPR proteins provide a potentially reversible approach to knockdown mitochondrial genes by targeting RNA (Colas des Francs-Small et al. 2018). Different levels of knockdown effect can be achieved in T1 selection by using the designed PPR protein (Figs. 2 and 3). Those phenotypes can be transmitted through generations after T1 selection (Fig. 1C), providing versatile materials for laboratory research or breeding applications (Supplementary Figs. S3 and S5). The use of inducible or tissue-specific promoters would help modulate the effects of the transgene further, which is essential to study vital functions such as ATP production.

### PPR protein design for organelle biotechnology

The PPR code (Barkan et al. 2012; Miranda et al. 2018; Yan et al. 2019; Bernath-Levin et al. 2021) describing sequence-specific binding ability between PPR proteins and their organelle target RNAs makes custom design usable for organelle biotechnology (Colas des Francs-Small et al. 2018). Despite not being able to confirm in vitro binding by gel shift due to aggregation of the recombinant protein, our in planta results show that an engineered PPR RFL protein can bind and induce cleavage of a new target transcript within the coding sequence of *atp1*. Recently, engineered PPR10 proteins in combination with their RNA targets were used to activate plastid transgenes (Rojas et al. 2019), resulting in a ∼40-fold increase in accumulation of the foreign proteins. That research established a method using chloroplasts as biofactories to synthesize and store valuable biological molecules. With more natural PPR proteins being functionally validated, the artificially designed PPR proteins have the potential to expand their application in organelles (Bernath-Levin et al. 2021; Royan et al. 2021).

## Materials and methods

### Protein designing, gene cloning, and transformation

The designed RPF2-*atp1* gene with a 3 × FLAG tag in C-terminal and a fragment containing the NOS promoter and the coding sequence of the 25 amino acid potato (*Solanum tuberosum*) FDH mitochondrial targeting peptide (Colas des Francs-Small et al. 1993) were commercially synthesized and cloned between the *Eco*RI and *Bam* HI sites of pCAMBIA1380 binary vector (Bevan 1984) by Gibson assembly. The synthetic genes were transferred to *A. tumefaciens* and introduced into *Arabidopsis* (*A. thaliana*) plants using the floral dip method (Clough and Bent 1998).

### Primary mutant screening

For primary mutant screening, 32 T1 RPF2-*atp1* plants were grown in chambers with WT plants, plants expressing native RPF2, and the Complex V-deficient editing mutant *opt87* (Hammani et al. 2011) as controls. All plants were grown under a 16-h light/8-h dark photoperiod throughout this study unless otherwise specified. Genomic PCR was performed with primers specific for the construct (FDHPre3F and RPF2 410R) (Supplementary Table S10).

### Total RNA extraction and northern blotting

For an early screen in T1 generation, total RNA was extracted from a single leaf of 4-wk-old plants (harvested in the middle of the day) using PureZol (Bio-Rad). Eight micrograms of total RNA were run on a 1.2% denaturing agarose gel and transferred onto the Hybond N + membrane (Amersham). Northern blotting was performed as described previously (Baudry et al. 2022) using oligonucleotide probes labeled at the 5′-end with biotin (Supplementary Table S10). The membranes were prehybridized for 1 to 2 h at 50 °C in 5 × SSC, 7% *w*/*v* SDS, 100 *μ*g·ml^−1^ heparin, 20 mM Na_2_HPO_4_ (pH 7.5) and hybridized overnight in the same buffer containing 1 nM biotinylated probe. Three short washes in 3 × SSC, 5% *w*/*v* SDS, 25 mM Na_2_HPO_4_ pH 7.5 were performed at room temperature. The northern blots were developed with the Pierce Chemiluminescent Nucleic Acid Detection Module Kit (Thermo Fisher Scientific).

### Mitochondrial RNA extraction and cRT-PCR

Crude preparations of mitochondria were isolated from 3-wk-old Col-0 and RPF2-*atp1* seedlings sown densely on half-strength MS plates as previously described (Colas des Francs-Small et al. 2012). Mitochondrial RNA was purified from the mitochondrial pellets using PureZol (Bio-Rad), and for each reaction, 2 to 3 *μ*g were treated with Turbo DNase (Ambion). RNA was circularized using T4 RNA ligase and reverse transcription was performed with the Superscript III (Invitrogen) using specific primers (*atp1* RT-407R for the full transcript or the fragment upstream of the cleavage site and *atp1* RT5BR for the fragment downstream of it). PCR was performed with a nested primer (*atp1* cRT-195R or cRT-5DR) and a specific forward primer (*atp1* cRT-2F or *cRT-3F*) (Supplementary Table S10). PCR products were sequenced by Macrogen (macrogen.com) and the sequences aligned in Geneious Prime (2020.0.4).

### RT-qPCR

Total RNA was isolated from 2-wk-old seedlings grown on plates (collected in the middle of the day) using RNAzol reagent (Sigma-Aldrich, Merck) and DNA precipitation with a 4-fold volume of bromoanisole. Reverse transcription was performed on 1 *μ*g of RNA using random hexamers as previously described (Baudry et al. 2022).

The primers used are detailed in Supplementary Table S10.

### Protein electrophoresis and western blotting

BN–PAGE, SDS–PAGE, and western blotting were performed as previously described (Colas des Francs-Small et al. 2014; Vincis Pereira Sanglard and Colas des Francs-Small 2022). The antibodies used in this work are listed in Supplementary Table S11.

### RNA sequencing

Total RNA was isolated with PureZol reagent (Bio-Rad) from central rosette leaves and emerging flower buds of 6-wk-old Col-0 and RPF2-*atp1*-9 plants (grown in soil and collected in the middle of the day). Three independent libraries for each genotype were made from total RNA treated with 250 ng of Turbo DNase (Ambion) using an Illumina TruSeq Stranded library preparation kit with Ribo-Zero plant and random-primed reverse transcriptase. Sequencing (150 bp, paired ends) was performed on an Illumina HiSeq4000 sequencer by Novogene (Novogene.com). RNA-seq data analysis methods are described in the Supplemental experimental procedures.

### Proteomic analysis

For quantitative untargeted MS, crude mitochondrial pellets were obtained from 3-wk-old WT, RPF2 native, RPF2-*atp1-9*, and RPF2-*atp1-16* seedlings densely sown on plates and the samples from 3 independent experiments were prepared as previously described (Colas des Francs-Small et al. 2014; Petereit et al. 2020). Samples were analyzed by LC-MS on a Thermo Exploris 480 mass spectrometer using data-dependent acquisition (see the Supplemental experimental procedures).

### Adenylate measurements

Absolute quantitation of AMP, ADP, and ATP from 3-wk-old seedling aerial parts or roots grown on plates by LC-MS was carried out according to (Straube et al. 2021) with slight modifications (see the Supplemental experimental procedures).

For the ATP synthesis rate, 20 *µ*g of purified mitochondria isolated from 3-wk-old native RPF2, RPF2 *atp1*-9, and RPF2 *atp1*-16 seedlings were equilibrated in 200 *µ*l of respiration buffer (Meyer et al. 2009) containing 2 mM ADP. The respiration reactions (triplicates) were started by the addition of NADH (1 mM final concentration) and stopped after 5 min by the addition of 15% *w*/*v* TCA. The adenylates were subsequently quantified according to the method described above. These samples were diluted 1/20 and the injection volume was 1 *µ*l.

### MS analyses of organic acids and amino acids

Three-week-old seedlings (∼25 mg) grown on plates were collected at the end of the night and snap frozen in liquid nitrogen. Metabolites were extracted as previously specified (Lee et al. 2021). For LC-MS analysis of organic acids, sample derivatization was carried out based on a previously published method with modifications (Han et al. 2013). Samples were analyzed by an Agilent 1100 HPLC system coupled to an Agilent 6430 Triple Quadrupole (QQQ) mass spectrometer equipped with an electrospray ion source as described previously (Lee et al. 2021). For amino acid quantification, dried samples were resuspended in 50-ml water and analyzed as described in Le et al. (2021).

### Accession numbers

The RNA-seq data have been deposited in the BioProject database, under the accession numbers PRJNA768306 (WT) and PRJNA893436 (RPF2-*atp1*-9). The AGI numbers of the genes/transcripts/proteins mentioned in this work are given in Supplementary Table S2.

## Supplementary Material

kiae008_Supplementary_Data

## Data Availability

Quantitative untargeted MS data have been deposited to the ProteomeXchange Consortium via the PRIDE (Perez-Riverol et al. 2022) partner repository with the data set identifier PXD037659.

## Supplementary data

The following materials are available in the online version of this article.

**Supplementary Figure S1.** Annotated sequence of the RPF2-*atp1* protein.

**Supplementary Figure S2.** Integration and expression of the RPF2-*atp1* construct in transgenic plants.

**Supplementary Figure S3.** Phenotypic observation of the RPF2-*atp1* transgenic lines.

**Supplementary Figure S4.** Trait investigation of 4 RPF2-*atp1* transgenic lines.

**Supplementary Figure S5.** Night respiration rates are increased in the RPF2-*atp1* plants.

**Supplementary Figure S6.** Abundances of mitochondrial transcripts in WT and RPF2-*atp1* plants.

**Supplementary Figure S7.** Splicing of most mitochondrial introns is higher in WT plants than in RPF2-*atp1* plants.

**Supplementary Figure S8.** The apparent editing rate at most mitochondrial editing sites is higher in WT plants than in RPF2-*atp1* plants.

**Supplementary Figure S9.** Relative transcript (from RNA-seq) and protein abundances.

**Supplementary Figure S10.** Adenylate levels are similar in WT and RPF2-*atp1* plants, but ATP synthesis rates are considerably lower in RPF2-*atp1* plants.

**Supplementary Figure S11.** Variations in leaf organic acid and amino acid contents in WT and RPF2-*atp1* plants.

**Supplementary Table S1.** Subunit composition of the mitochondrial ATP synthase in *Arabidopsis*.

**Supplementary Table S2.** Abundance of transcripts and peptides of respiratory complex subunits.

**Supplementary Table S3.** GO terms of transcripts significantly downregulated in RPF2-*atp1*-9 as compared with WT.

**Supplementary Table S4.** GO terms of transcripts significantly upregulated in RPF2-*atp1*-9 as compared with WT.

**Supplementary Table S5.** Metabolomic data and statistical analyses.

**Supplementary Table S6.** Root adenylate measurements, average ATP/ADP ratios, and adenylate charge.

**Supplementary Table S7.** Sum of absolute amino acid abundances by families in nmol/g FW (and as a percentage of total amino acids).

**Supplementary Table S8.** Glycine to Ser ratios.

**Supplementary Table S9.** Upregulation and downregulation of genes encoding amino acid synthesis and degradation pathway enzymes in RPF2-*atp1* compared to Col-0.

**Supplementary Table S10.** Primers used in this work.

**Supplementary Table S11.** Antibodies used in this work.

## Dive Curated Terms

The following phenotypic, genotypic, and functional terms are of significance to the work described in this paper:

FDH Gramene: AT5G14780
FDH Araport: AT5G14780

AOX1A Gramene: AT3G22370
AOX1A Araport: AT3G22370

AOX1D Gramene: At1g32350
AOX1D Araport: At1g32350

NDA1 Gramene: AT1G07180
NDA1 Araport: AT1G07180

NDB4 Gramene: AT2G20800
NDB4 Araport: AT2G20800

ATP CHEBI: CHEBI:15422

ANAC017 Gramene: AT1G34190
ANAC017 Araport: AT1G34190

HSP70 Gramene: AT3G12580
HSP70 Araport: AT3G12580

NDB2 Gramene: AT4G05020
NDB2 Araport: AT4G05020

ATP4 Gramene: ATMG00640
ATP4 Araport: ATMG00640
