# Supplementary material for: Mitochondrial atp1 mRNA knockdown by a custom-designed pentatricopeptide repeat protein alters ATP synthase
Source: Plant Physiol. 2024 Jan 11;194(4):2631–47. doi: 10.1093/plphys/kiae008 (PMC10980415; doi:10.1093/plphys/kiae008)
Supplement: kiae008_Supplementary_Data [file kiae008_supplementary_data.zip › PP2023RA01300DR1_Supplemental_Data.pdf]

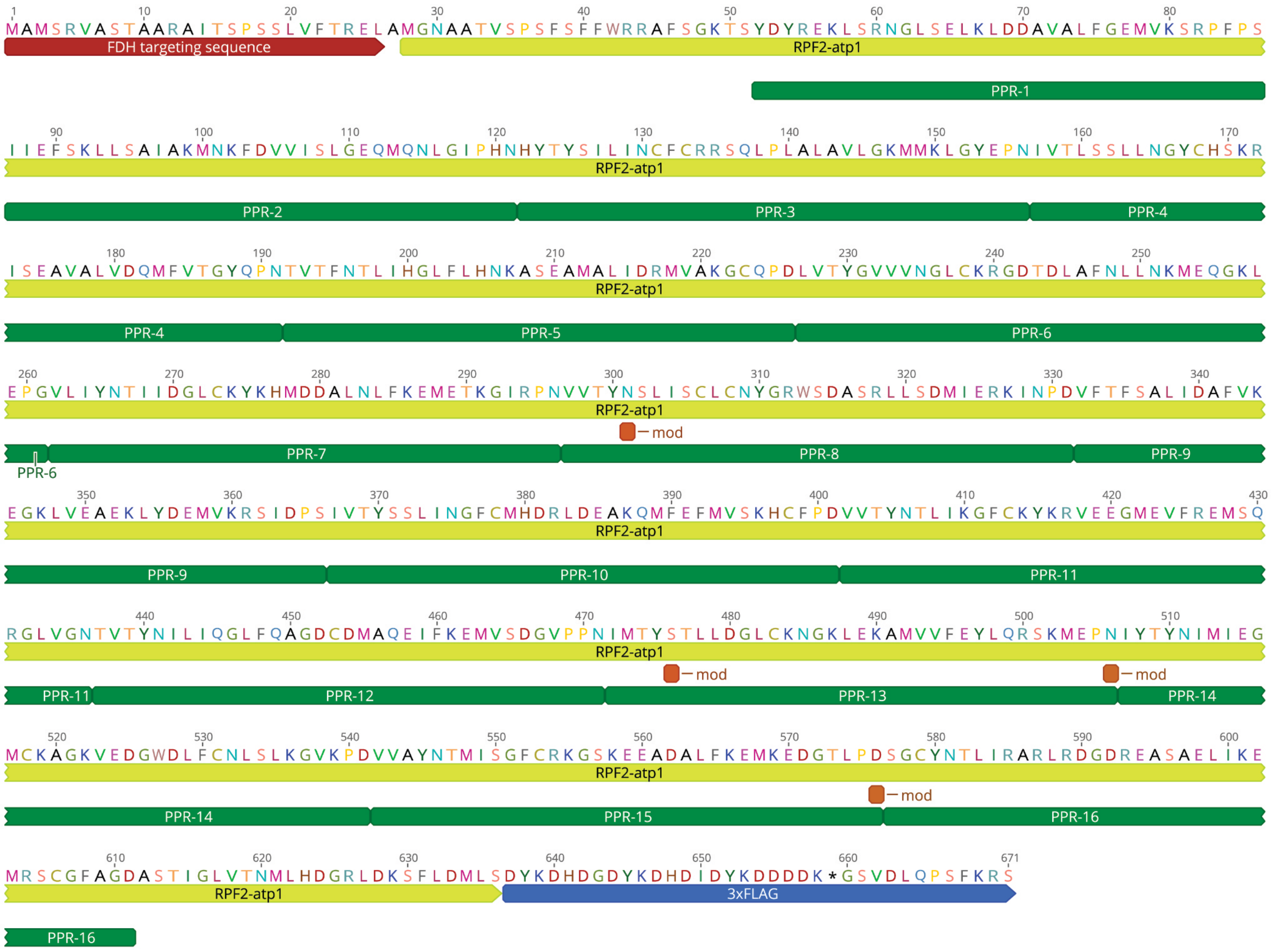

Supplemental Figure S1. Annotated sequence of the RPF2-*atp1* protein. RPF2-*atp1* expression was driven by the nopaline synthase promoter. The RPF2-*atp1* protein was targeted to mitochondria by the formate dehydrogenase targeting peptide (FDH TS, in red). The residues modified from the native RPF2 protein are marked in red, the PPR motifs in green and the 3xFLAG tag is in dark blue. The diagram was prepared in Geneious Prime 2020.1.

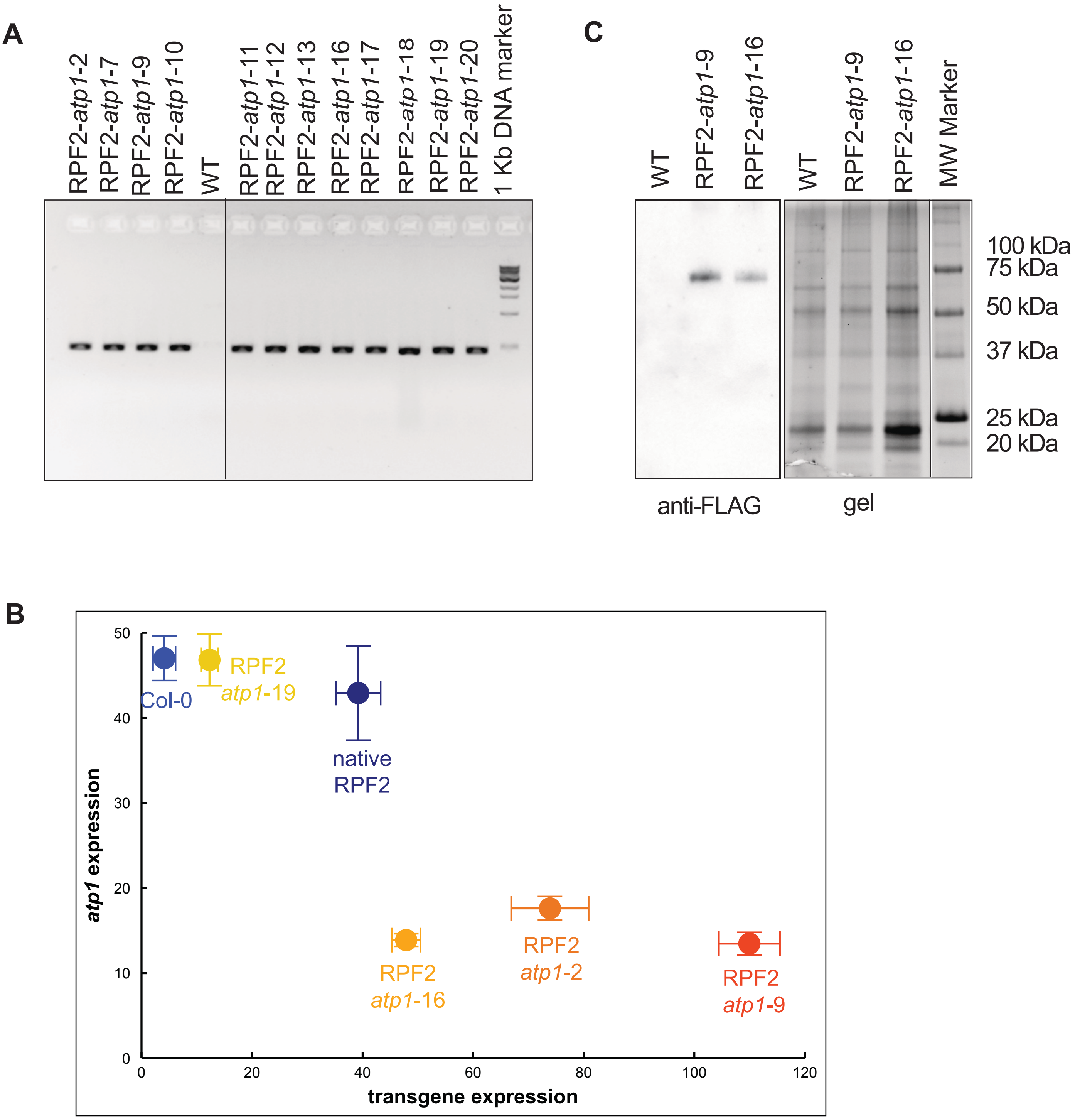

Supplemental Figure S2. Integration and expression of the RPF2-*atp1* construct in transgenic plants. (**A**) PCR verification of the integration of the RPF2-*atp1* construct in transgenic plants (T1 generation). (**B**) Relative expression levels of RPF2 transgenes and *atp1* in different transgenic lines compared to wild-type. Relative amounts of transcripts (in arbitrary units) were measured by RT-qPCR using specific primer pairs for RPF2 transgenes and for mitochondrial *atp1* transcripts. Five mitochondrial transcripts (*nad1*, *nad2*, *nad5*, *rpl2* and *rps4*) were used as internal controls for normalisation between samples. Error bars indicate standard error of the mean of technical repeats (n=9). (**C**) Western blot (left panel) showing FLAG tag expression in the RPF2-*atp1*-9 and RPF2-*atp1*-16 transformants. The corresponding SDS PAGE gel is shown in the right panel.

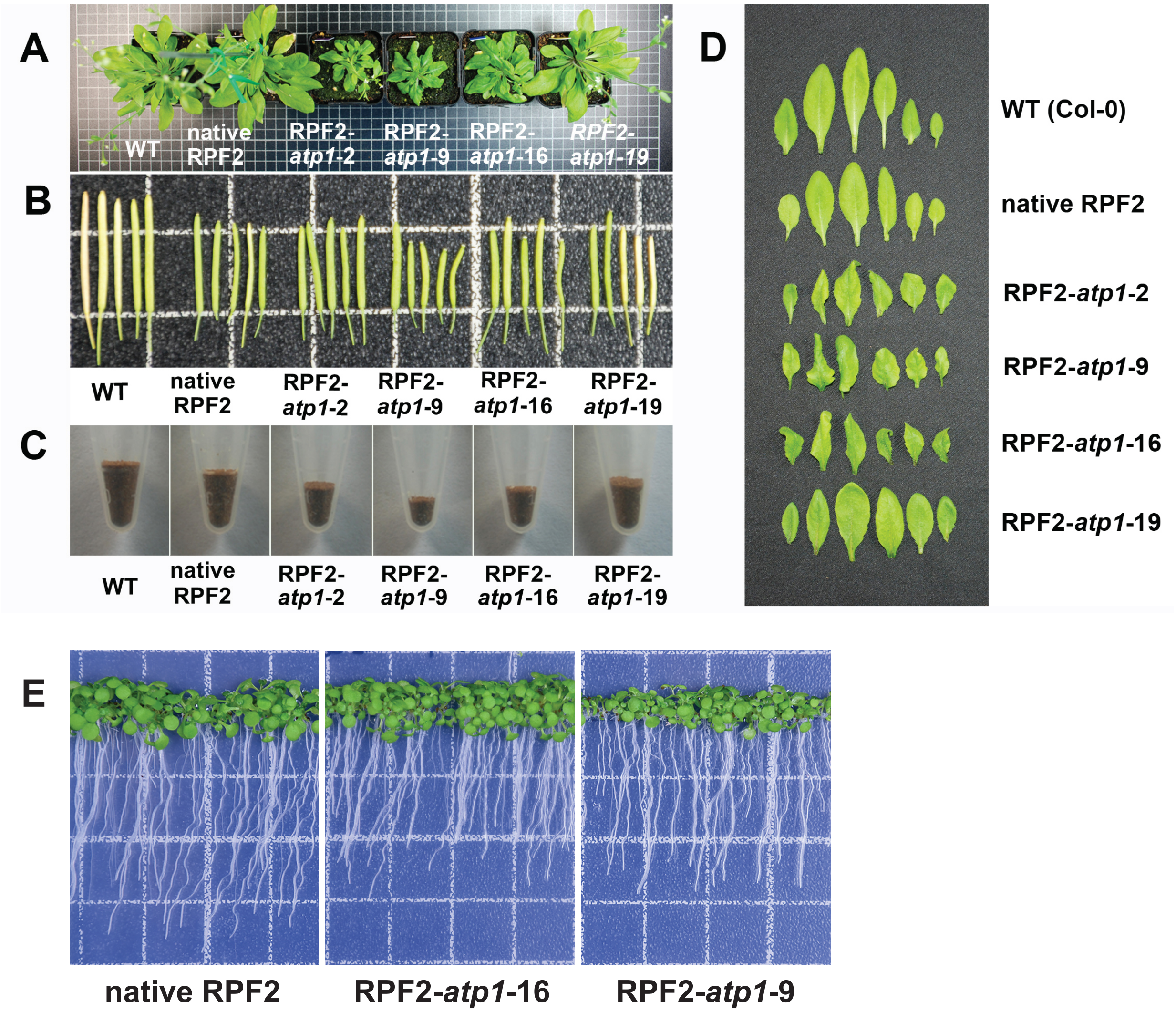

Supplemental Figure S3: Phenotypic observation of the RPF2-*atp1* transgenic lines. (**A**) Phenotypes of 6-week-old plants grown under 16-hour photoperiod, (**B**) Silique length, (**C**) Seed production per plant, (**D**) Detached leaves from 6-week-old plants, (**E**) Root lengths of 10-day-old seedlings.

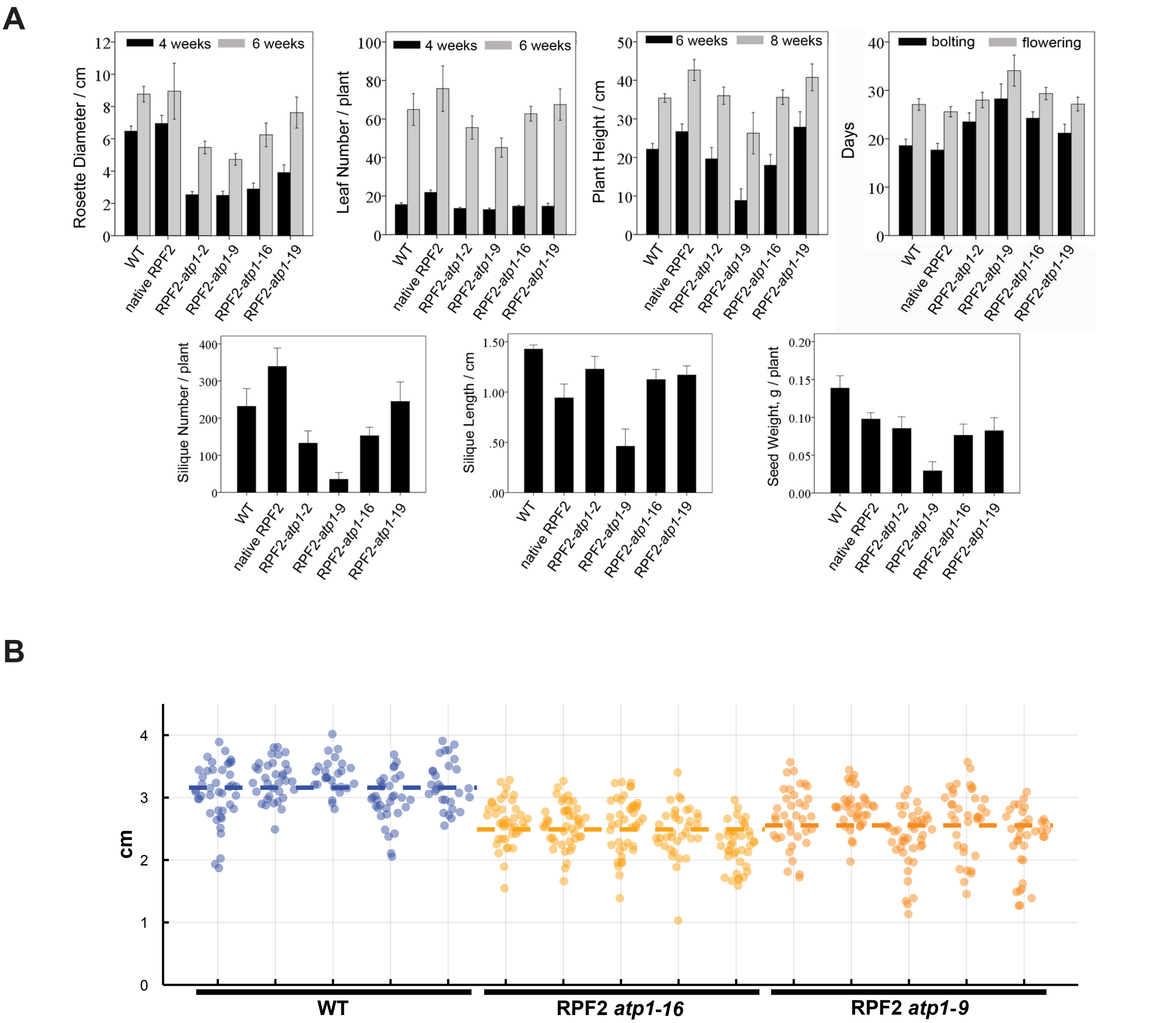

Supplemental Figure S4: Trait investigation of four RPF2-*atp1* transgenic lines. (**A**) Rosette diameter, leaf number and plant height were measured on 4- and 6-week-old plants. The number of days necessary for bolting and flowering in all genotypes were noted. Silique numbers per plant, silique length and seed weight per plant were recorded for all genotypes. Error bars indicate standard error of the mean of technical repeats (n=12).

(**B**) The root lengths of 10-day-old seedlings were measured using the Fiji *ImageJ* software on 5 plates containing about 40 seedlings each for native RPF2, RPF2-*atp1*-16 and RPF2-*atp1*-9 lines in T4 generation. The seedlings were grown on vertical plates under a16-hour photoperiod. Primary root tips were identified and marked manually in the images; root lengths were estimated as the linear distance between the root tip and the base line where the seeds were placed. Dashed lines represent the mean length and root lengths are significantly different between control and RPF2-*atp1* lines (p = 7.5 10^{-44} for RPF2-*atp1*-16; p = 1.1 10^{-29} for RPF2-*atp1*-9, using equal variance *t*-test).

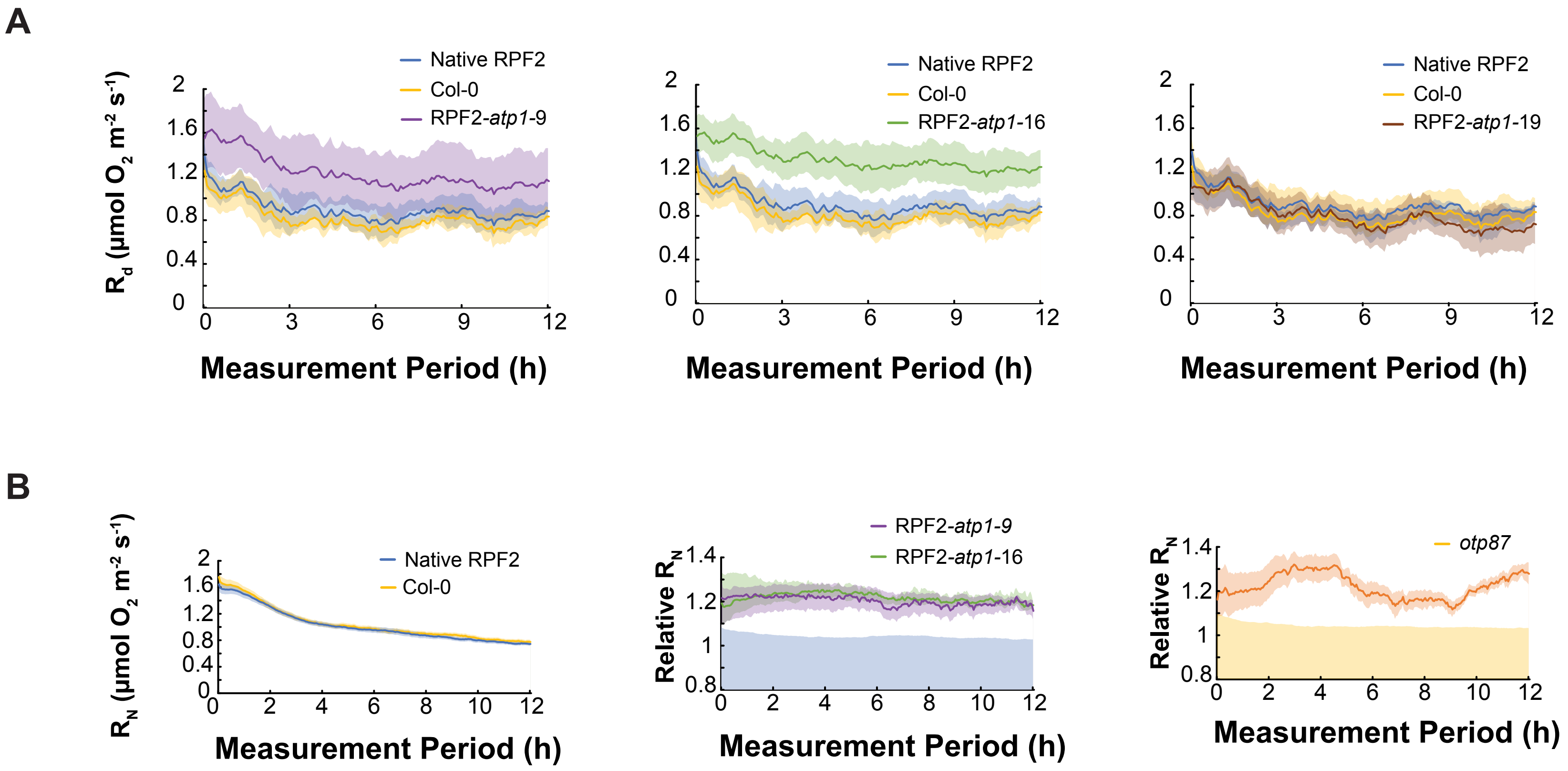

Supplemental Figure S5. Night Respiration rates are increased in the RPF2-*atp1* plants. Traces represent moving averages **(A)** Rd (n = 4) and **(B)** R_N (n ≥ 16) of whole mature leaves of WT, native RPF2, *otp87* and RPF2-*atp1* transgenic lines. Shaded areas represent 95% confidence intervals. Relative R_N rates of *otp87* was calculated relative to Col-0 (yellow shaded area). Relative R_N rates of RPF2-*atp1*–9 and RPF2-*atp1*–16 were calculated relative to native RPF2 (blue shaded area).

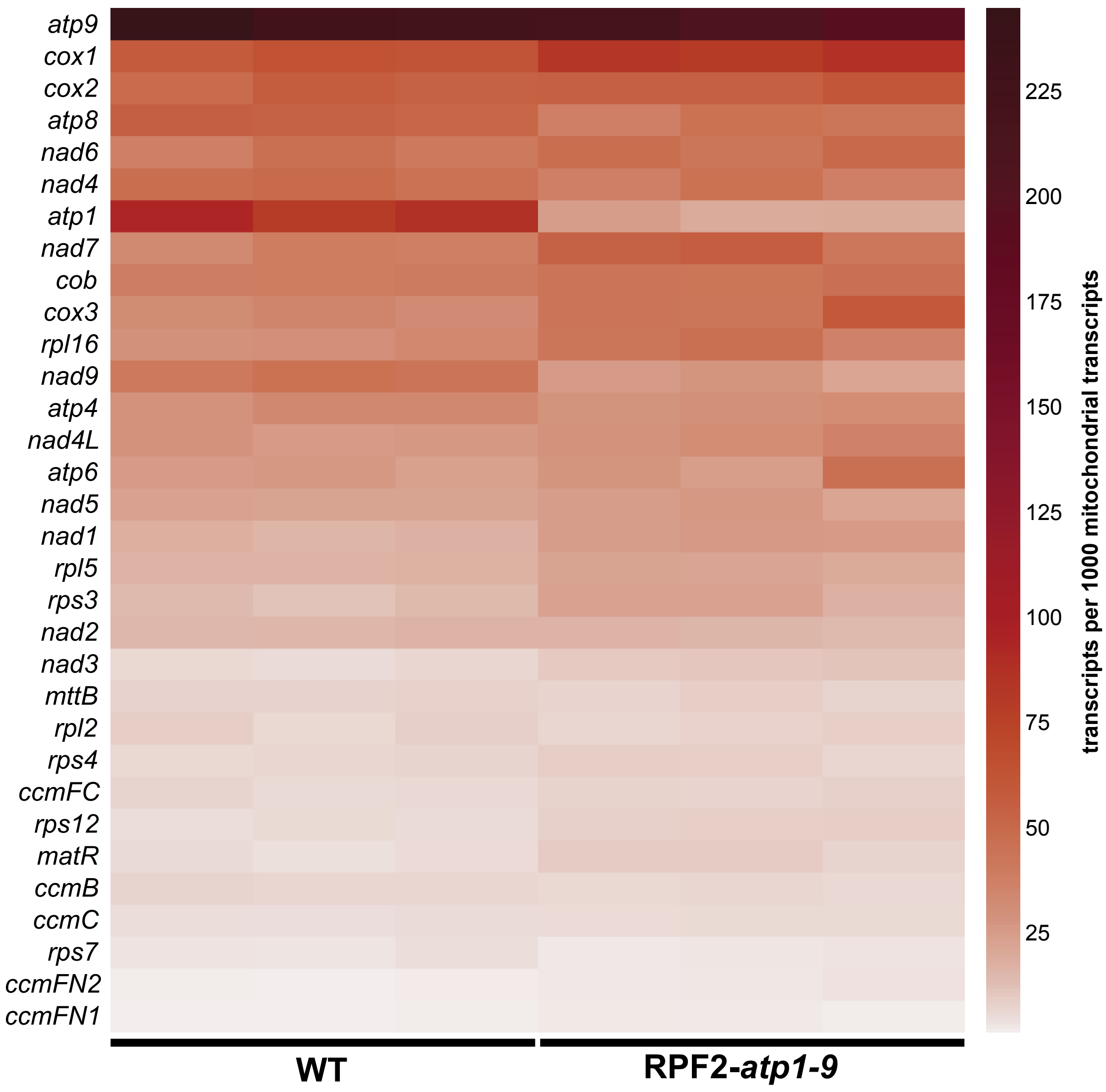

Supplemental Figure S6. Abundances of Mitochondrial Transcripts in WT and RPF2-*atp1* Plants. RNA-seq reads from WT and RPF2-*atp1*-9 samples were mapped to the mitochondrial genome and the read coverage was normalised using the method of DESeq2 and by transcript length. The transcript abundances are visualised as the number of transcripts per thousand mitochondrial transcripts. Three WT and three RPF2-*atp*1-9 samples were analysed.

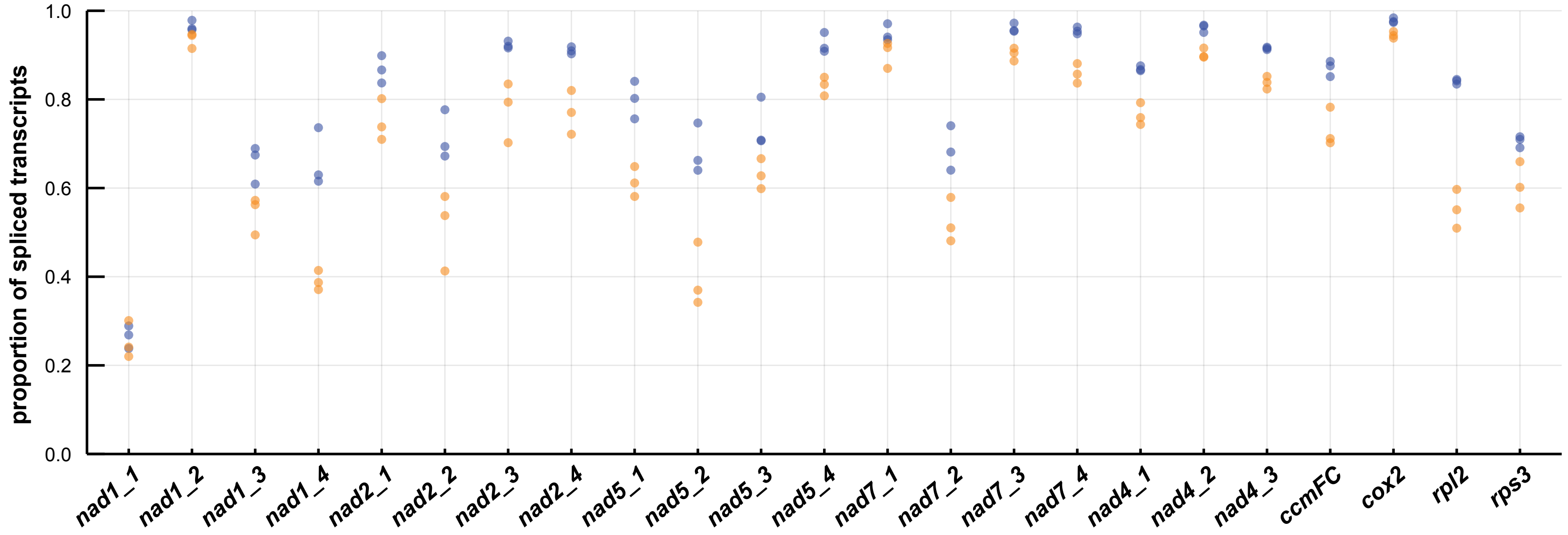

Supplemental Figure S7. Splicing of most mitochondrial introns is higher in WT plants than in RPF2-*atp1* plants. A dotplot showing the proportion of spliced transcripts for all the mitochondrial introns, as calculated from RNA-seq reads. Blue markers indicate WT samples, orange markers indicate RPF2-*atp1*-9 samples. Three independent samples are shown for each genotype.

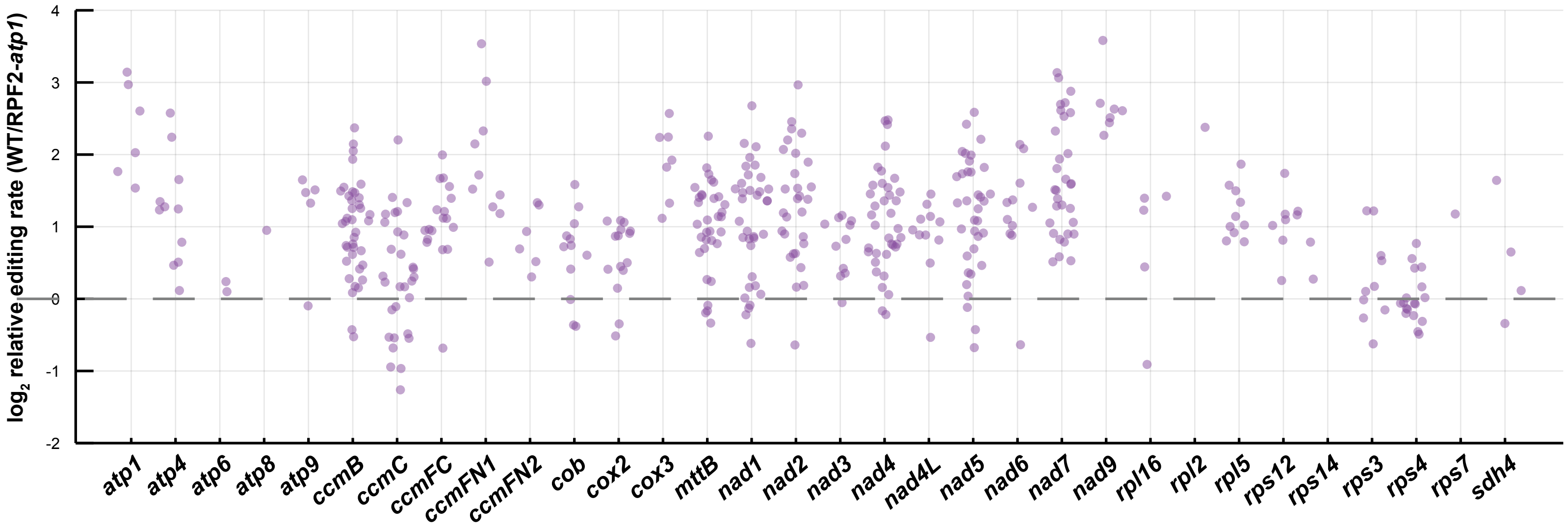

Supplemental Figure S8. The apparent editing rate at most mitochondrial editing sites is higher in WT plants than in RPF2-*atp1* plants. A grouped dot plot showing the apparent relative editing rate (WT/RPF2-*atp1*) for 461 editing sites within coding sequences. Relative editing rate was calculated as the odds ratio (WT/RPF2-*atp1*), where the odds were calculated in each case as the proportion of edited transcripts/proportion of unedited transcripts, as calculated from RNA-seq reads. The odds were the means of 3 samples in each case. The dashed line indicates equal apparent rate in both genotypes.

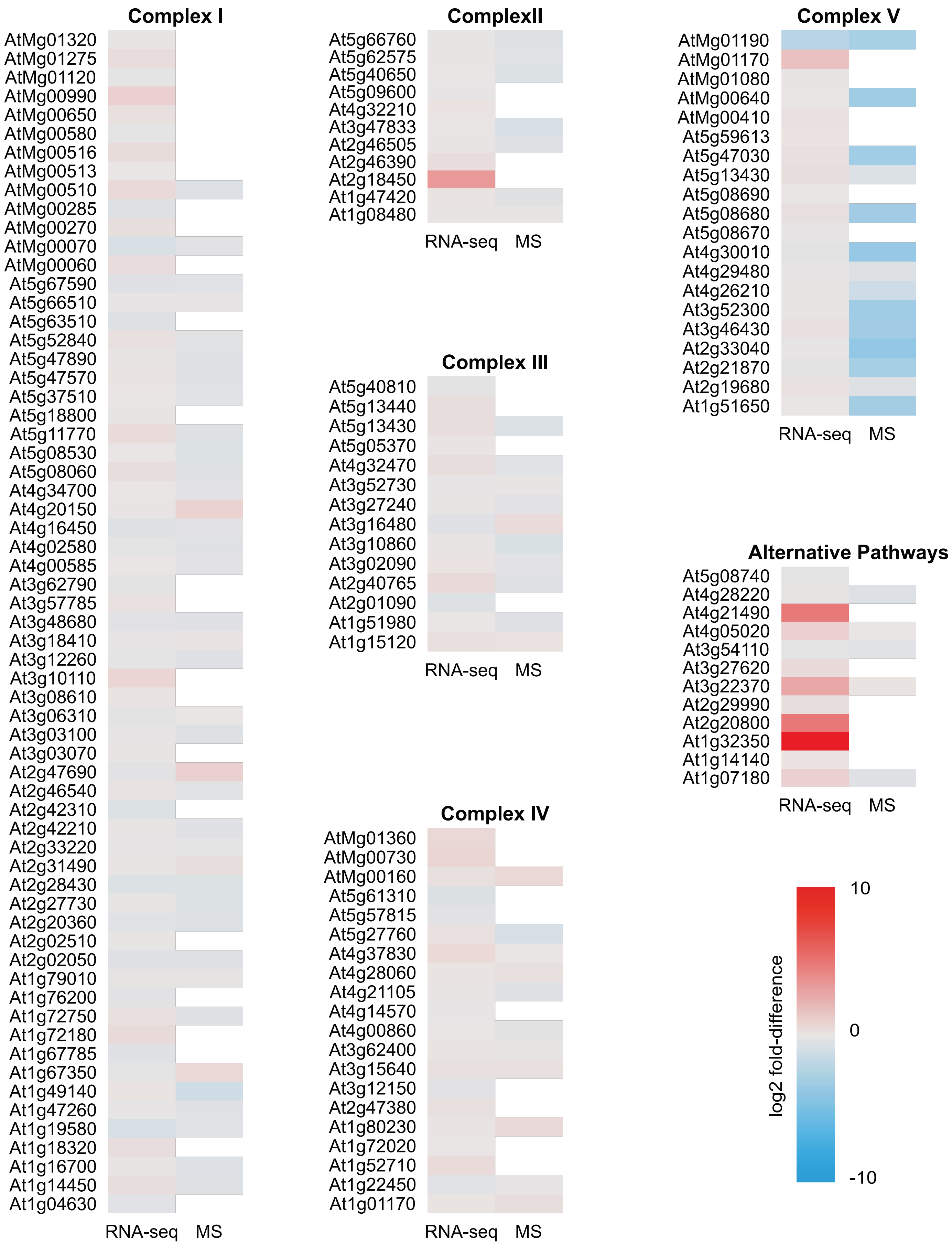

Supplemental Figure S9. Relative transcript (from RNA-seq) and protein abundances (as estimated by quantitative untargeted mass spectrometry (MS)). The heat maps show data for transcripts and proteins from the respiratory complexes I-V and alternative pathways. RNA-seq data are from WT and RPF2-*atp1*-9 (3 repeats each) and MS data are from 8 RPF2-*atp1* samples and 8 phenotypically WT samples (4 from WT plants, 4 from plants expressing the native RPF2). The colour chart indicates log2-fold differences between WT and RPF2-*atp1* plants. Blank rows indicate missing values in the MS data.

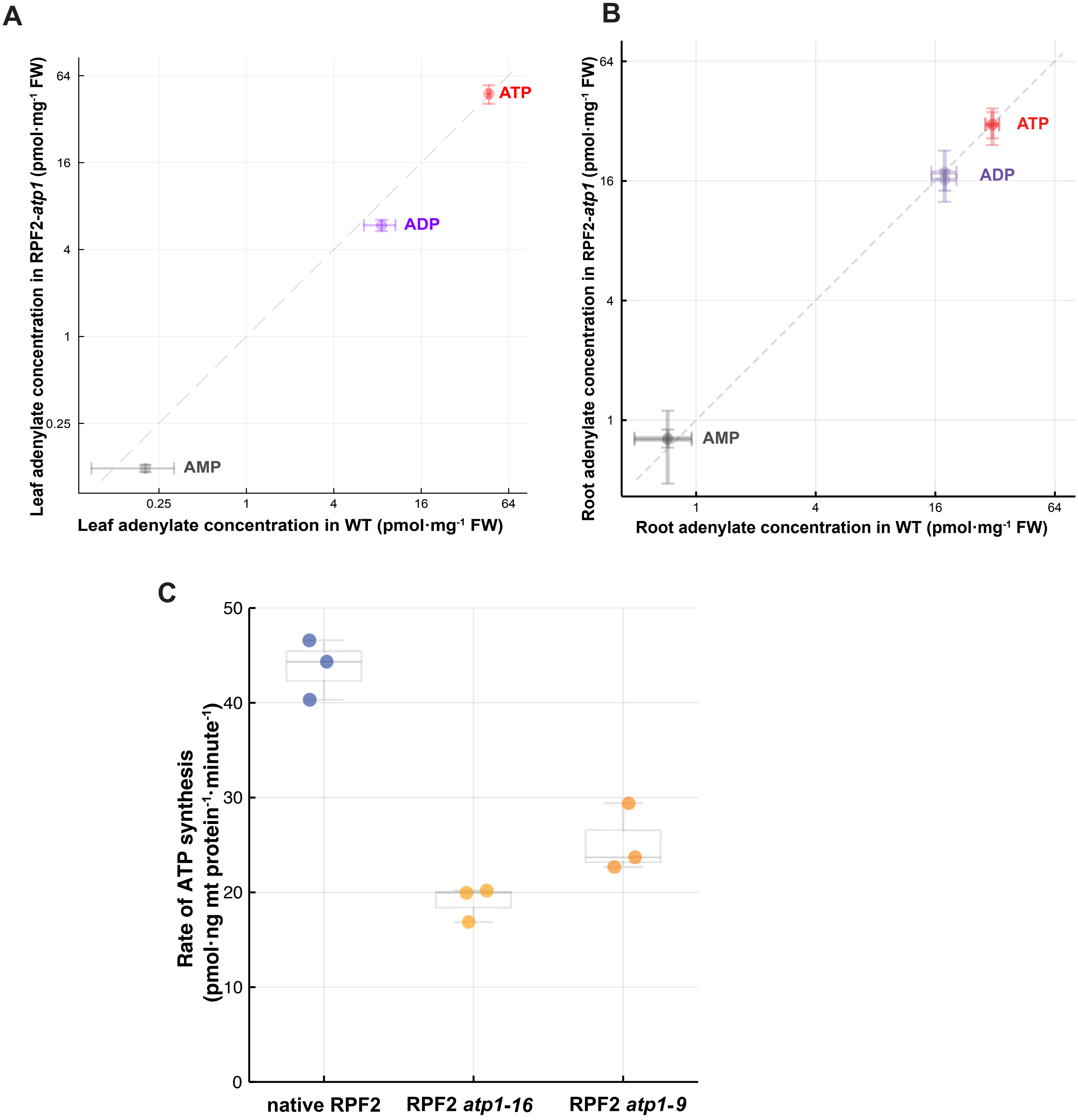

Supplemental Figure S10. Adenylate levels are similar in WT and RPF2-*atp1* plants but ATP synthesis rates are considerably lower in RPF2-*atp1* plants. Total leaf (**A**) and root (**B**) adenylates in RPF2-*atp1* plants as compared to WT. The dashed diagonal line indicates equal concentrations in both genotypes. Error bars indicate standard deviation of the mean of technical repeats (n=2 and n=5 respectively). (**C**) ATP synthesis rates were determined by LC/MS in isolated mitochondria from WT (blue), RPF2-*atp1*-16 (yellow) and RPF2-*atp1*-9 (orange). The boxplot center line is the median; box limits are the upper and lower quartiles; whiskers represent 1.5x interquartile range; points are the measured rates (n=3).

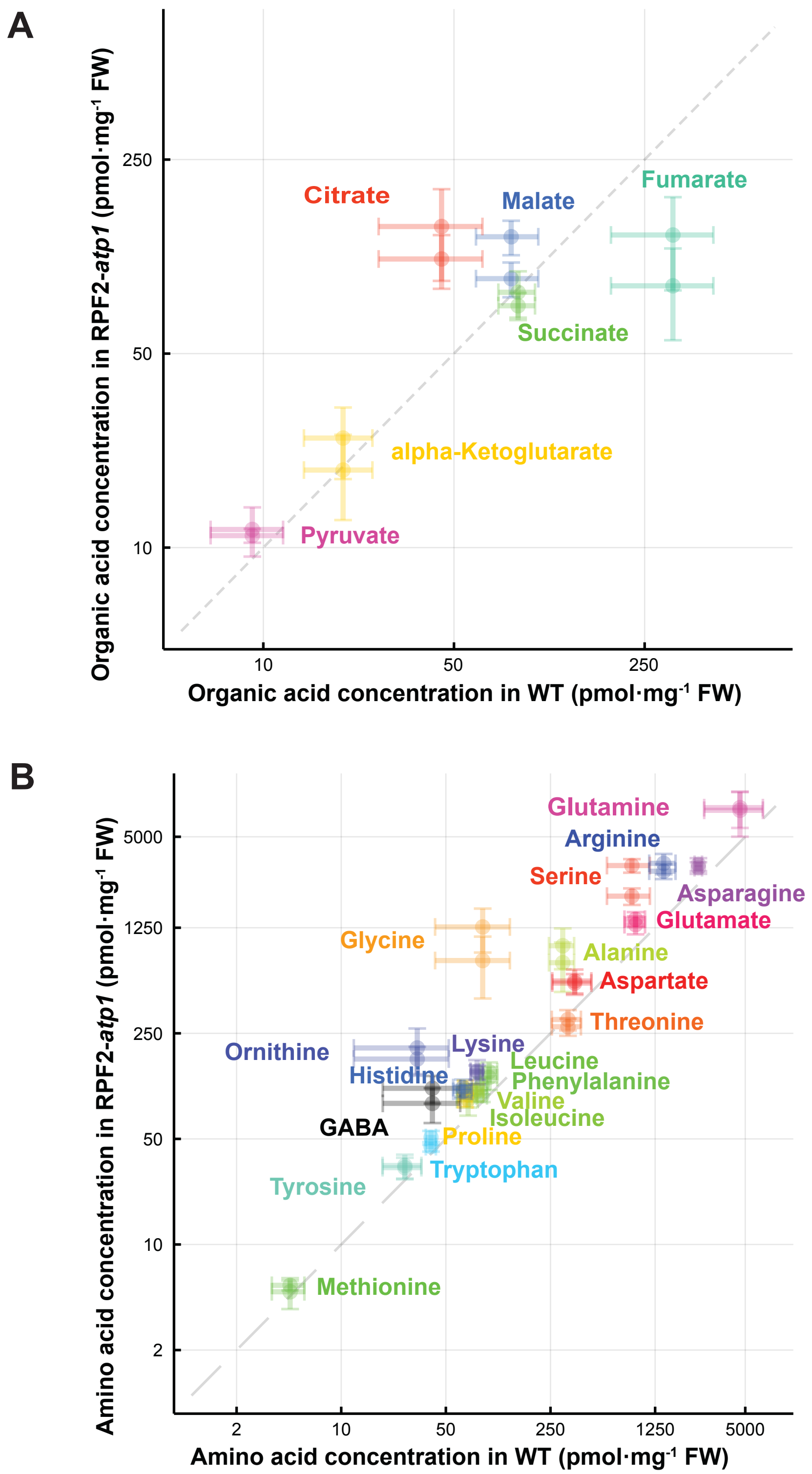

Supplemental Figure S11. Variations in leaf organic acid and amino acid contents in WT and RPF2-*atp1* plants. The graphs show organic acid (**A, C**) and amino acid (**B, D**) concentrations in RPF2-*atp1*-9 *and* RPF2-*atp1*-16 as compared to WT. (**A**, **B**) Dots on the diagonal indicate equal concentrations in all genotypes. The x and y axes are log scales. Error bars indicate standard deviation of the mean of technical repeats (n=5).

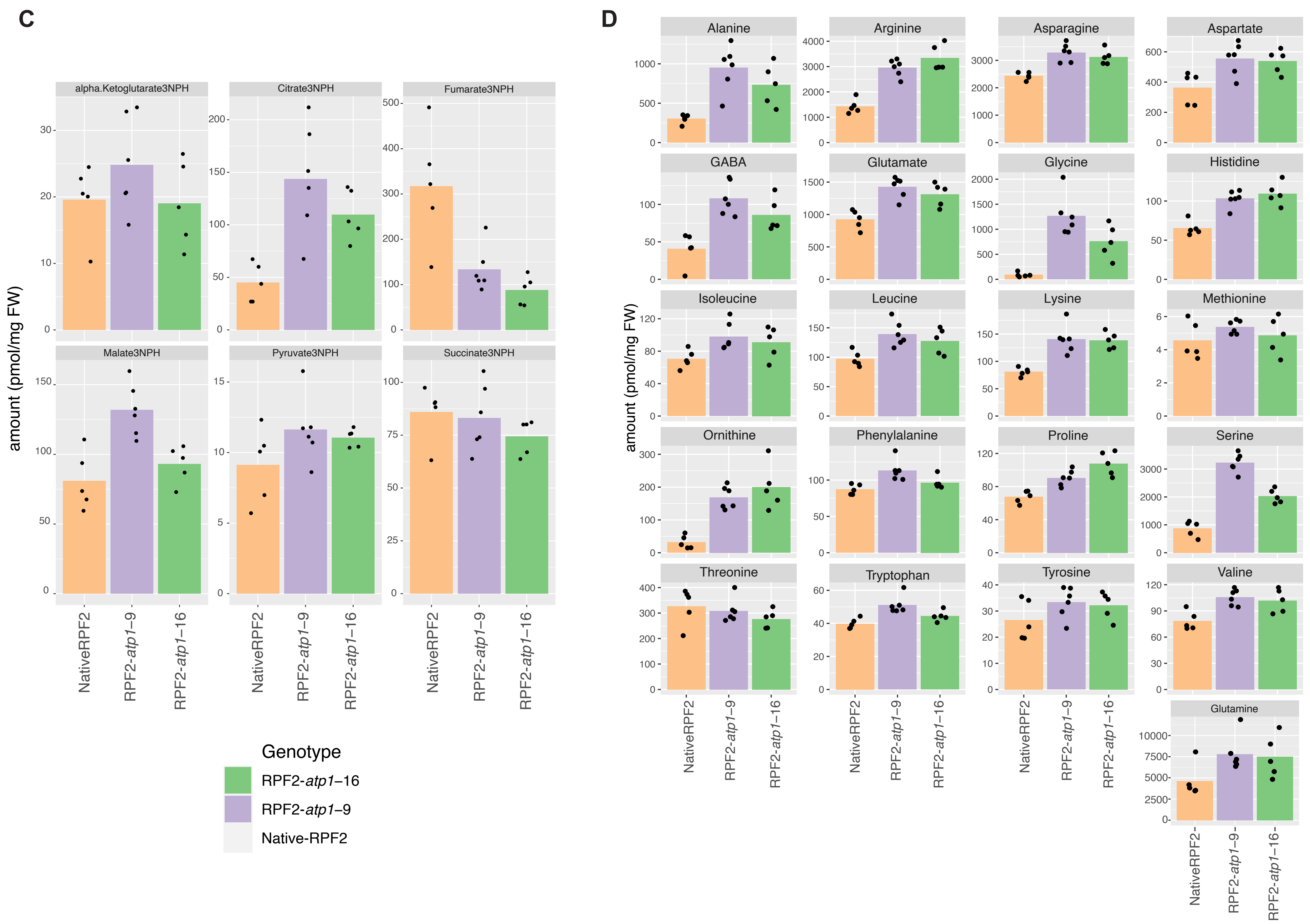

C
D
alpha.Ketoglutarate3NPH
Citrate3NPH
Fumarate3NPH
Malate3NPH
Pyruvate3NPH
Succinate3NPH
amount (pmol/mg FW)
NativeRPF2
RPF2-atp1–9
RPF2-atp1–16
Genotype
RPF2-atp1–16
RPF2-atp1–9
Native-RPF2
Alanine
Arginine
Asparagine
Aspartate
GABA
Glutamate
Glycine
Histidine
Isoleucine
Leucine
Lysine
Methionine
Ornithine
Phenylalanine
Proline
Serine
Threonine
Tryptophan
Tyrosine
Valine
Glutamine

Supplemental Table S1. Subunit composition of the mitochondrial ATP Synthase in Arabidopsis.

ATPase domain	Subunit	Gene	AGI number	Genome	Proteomics study
**F_1 head**	α	*atp1*	AtMg01190	mitochondrial	y
	β	*ATP2*	At5g08670<br>At5g08680<br>At5g08690	nuclear	y
**F_1 central stalk**	γ	*ATP3*	At2g33040	nuclear	y
	δ		At5g47030	nuclear	y
	ε		At1g51650	nuclear	y
**F_o motor**	a	*atp6*	AtMg00410	mitochondrial	n
	c	*atp9*	AtMg01080	mitochondrial	n
**F_o peripheral stalk**	b	*atp4*	AtMg00640	mitochondrial	
	d	*ATPD*	At3g52300	nuclear	y
	OSCP	*ATP5*	At5g13450	nuclear	y
	8	*atp8*	AtMg00480	mitochondrial	n
	f		At4g30010	nuclear	y
	F_Ad	*MGP1*	At2g21870	nuclear	y
	i/j, 6-kD		At3g46430,<br>At5g59613	nuclear	y
**F_o dimerisation**	e		At5g15320	nuclear	y
	g		At2g19680<br>At4g29480	nuclear	y
**inhibitor**	IF1		At2g27730	nuclear	y
**Assembly factor**	ATP11	*P11*	At2g34050	nuclear	n
	ATP12	*P12*	At5g40660	nuclear	n
	ATP23	*AtATP23*	At3g03420	nuclear	n
	TMEM70		At2g35790	nuclear	n

Supplemental Table S2. Abundance of transcripts and peptides of respiratory complex subunits (Excel table).

Supplemental Table S3. GO terms of transcripts significantly down-regulated in RPF2-*atp1-9* as compared with WT.

GO.ID	Term	Annotated	Significant	Expected	weight01	Class
GO:0010584	pollen exine formation	45	43	5.84	< 1e-30	Male reproductive development
GO:0048481	plant ovule development	36	25	4.67	6.0e-15	Female reproductive development
GO:0048441	petal development	46	31	5.97	8.8e-15	Floral organ development
GO:0009827	plant-type cell wall modification	59	32	7.66	2.8e-14	Cell wall organisation
GO:0009860	pollen tube growth	62	32	8.05	1.8e-13	Male reproductive development
GO:0048443	stamen development	48	27	6.23	1.9e-11	Female reproductive development
GO:0019953	sexual reproduction	64	29	8.31	2.4e-11	Reproductive Process
GO:0010093	specification of floral organ identity	16	13	2.08	1.0e-09	Reproductive Process
GO:0009886	post-embryonic animal morphogenesis	13	11	1.69	9.8e-09	Post-embryonic development
GO:0006869	lipid transport	26	14	3.38	7.1e-07	Lipid transport
GO:0048507	meristem development	96	24	12.47	4.5e-06	Meristem development
GO:0080110	sporopollenin biosynthetic process	5	5	0.65	3.6e-05	Male reproductive development
GO:0009556	microsporogenesis	13	8	1.69	5.4e-05	Male reproductive development
GO:0006508	proteolysis	121	21	15.71	6.5e-05	Protein metabolic process
GO:0048451	petal formation	24	11	3.12	8.1e-05	Reproductive Process
GO:0048453	sepal formation	24	11	3.12	8.1e-05	Reproductive Process

GO:0009718	anthocyanin-containing compound biosynth...	21	10	2.73	0.00012	Pigment biosynthetic process
GO:0000911	cytokinesis by cell plate formation	39	14	5.06	0.00022	Cell cycle
GO:0001708	cell fate specification	19	8	2.47	0.00050	Cell fate commitment
GO:0000226	microtubule cytoskeleton organization	42	12	5.45	0.00117	Organelle organisation

Supplemental Table S4. GO terms of transcripts significantly up-regulated in RPF2-*atp1-9* as compared with WT.

GO.ID	Term	Annotated	Significant	Expected	Weight	Class
GO:0009697	salicylic acid biosynthetic process	71	51	8.36	< 1e-30	Response to biotic stress
GO:0050832	defense response to fungus	99	60	11.66	6.1e-30	Response to biotic stress
GO:0010200	response to chitin	128	61	15.07	3.5e-25	Response to biotic stress
GO:0009867	jasmonic acid mediated signaling pathway	70	43	8.24	1.1e-23	Response to hormone stimulus
GO:0000165	MAPK cascade	63	40	7.42	7.2e-23	Response to stimulus
GO:0031348	negative regulation of defense response	77	44	9.07	2.1e-22	Response to stress
GO:0042742	defense response to bacterium	85	49	10.01	3.5e-22	Response to biotic stress
GO:0009862	systemic acquired resistance, salicylic ...	69	41	8.13	7.9e-22	Response to biotic stress
GO:0010363	regulation of plant-type hypersensitive ...	83	45	9.77	1.3e-21	Response to biotic stress
GO:0006612	protein targeting to membrane	84	45	9.89	2.5e-21	Protein targeting to membrane
GO:0010310	regulation of hydrogen peroxide metaboli...	51	34	6.01	1.4e-20	Regulation of cellular metabolic process
GO:0009627	systemic acquired resistance	121	73	14.25	3.4e-19	Response to biotic stress
GO:0009595	detection of biotic stimulus	35	28	4.12	4.2e-17	Response to biotic stress

GO:0034976	response to endoplasmic reticulum stress	96	48	11.31	8.0e-17	Response to stress
GO:0002679	respiratory burst involved in defense re...	45	27	5.30	7.8e-15	Response to stress
GO:0043069	negative regulation of programmed cell d...	48	27	5.65	7.2e-14	Regulation of cellular process
GO:0043900	regulation of multi-organism process	33	22	3.89	1.1e-13	Response to abiotic stimulus
GO:0002237	response to molecule of bacterial origin	30	20	3.53	1.6e-12	Response to biotic stress
GO:0009723	response to ethylene	85	35	10.01	1.8e-11	Response to hormone stimulus
GO:0042538	hyperosmotic salinity response	36	19	4.24	1.8e-09	Response to abiotic stimulus
GO:0009738	abscisic acid-activated signaling pathwa...	62	24	7.30	3.4e-08	Response to hormone stimulus
GO:0009611	response to wounding	71	26	8.36	3.5e-08	Response to stress
GO:0006865	amino acid transport	60	25	7.07	1.1e-07	Amino acid transport
GO:0009407	toxin catabolic process	48	19	5.65	6.4e-07	Response to chemical
GO:0009414	response to water deprivation	76	28	8.95	8.5e-07	Response to abiotic stimulus
GO:0030968	endoplasmic reticulum unfolded protein r...	46	18	5.42	1.6e-06	Response to unfolded protein
GO:0009695	jasmonic acid biosynthetic process	30	14	3.53	1.9e-06	Response to hormone stimulus
GO:0010167	response to nitrate	44	17	5.18	3.8e-06	Response to chemical
GO:0010583	response to cyclopentenone	40	16	4.71	4.3e-06	Response to chemical

Supplemental Table S5. Metabolomics Data and Statistics Analyses (Excel File). S5-1: Leaf adenylates; S5-2: Root adenylates; S5-3: Mitochondrial nucleotides; S5-4: Amino acids; S5-5: Glutamine; S5-6: Organic acids.

Supplemental Table S6. Root adenylate measurements, average ATP/ADP ratios and adenylate charge.

SAMPLE	AMP	ADP	ATP	ATP/ADP (average)	Adenylate charge
RPF2-atp1-9 R1	0.235901757	9.553578941	13.86151114		0.788056
RPF2-atp1-9 R2	0.231227659	7.021219522	10.54517571		0.789756
RPF2-atp1-9 R3	0.246429725	8.131770713	15.99138559		0.823045
RPF2-atp1-9 R4	0.284262018	4.713560707	9.513423652		0.818
RPF2-atp1-9 R5	0.294191061	4.826891588	9.118099299	1.765337	0.809846
RPF2-atp1-16 R1	0.305153275	9.980368419	14.4325364		0.78577
RPF2-atp1-16 R2	0.416602629	8.597589173	15.87873148		0.810573
RPF2-atp1-16 R3	0.324311947	9.383373768	15.60763754		0.801859
RPF2-atp1-16 R4	0.297030056	3.909612316	8.235761225		0.819019
RPF2-atp1-16 R5	0.413577844	6.697983858	13.39908742	1.812662	0.816555
WT1	0.316217643	7.040660358	14.87808796		0.827454
WT2	0.203345629	7.140021594	11.27062327		0.797284
WT3	0.261384818	7.582798341	13.15403888		0.806994
WT4	0.275248196	4.834197857	9.332092516		0.813569
WT5	0.473976472	5.668696013	11.50186683	1.87717	0.812502

Supplemental Table S7. Sum of absolute amino acid abundances by families in nmol/g FW (and as a percentage of total amino acids).

	WT (nmol/g FW)	RPF2-*atp1*-9	RPF2-*atp1*-16
Aromatic family	8.8 (2.1%)	10.6 (1.3%)	10.2 (1.3%)
Serine family	54.4 (12.8%)	240.1 (29.7%)	166.2 (21.1%)
Aspartate family	187.3 (44.1%)	234.0 (29%)	245.8 (31.3%)
Pyruvate family	27.5 (6.5%)	63.9 (7.9%)	58.1 (7.4%)
Glutamate family	146.3 (34.5%)	258.5 (32%)	305.8 (38.9%)
Total	424.2 (100%)	807.1 (100%)	786.1 (100%)

Supplemental Table S8. Glycine to serine ratios.

	Gly/Ser	*p*-value (T-test)
RPF2-*atp1*-9	0.397	0.0002
RPF2-*atp1*-16	0.387	0.0031
WT	0.102	

Supplemental Table S9. 36 Up- and down-regulation of genes encoding amino acid synthesis and degradation pathway enzymes in RPF2-*atp1* compared to Col-0 (Excel file).

Supplemental Table S10. Primers used in this work.

**Genotyping**	
FDH Pre3F	GGCGATGAGTCGTGTAGCTT
RPF2 410R	AGCGAAGAAAGCGTGACAAT
**RT-qPCR**	
PRE FDH2F	TGGCGATGAGTCGTGTAGCTTCT
RPF2 startR	ACAGTTGCAGCATTACCCATGGC
atp1-112683F	CTACAAGCCCTTAAAGGTGGATT
atp1-112878R	TCTCCCAGTATTGGAGACCTACT
atp1 cRT3F	GAGGTGCAAGGCTGACAGAAGTAC
*atp1 c*RT5DR	TTCAGGTTTRACACTATTTRGAATGGC
*nad1*a F	GACCAATAGATACTTCATAAGAGACCA
*nad1*a R	TTGCCATATCTTCGCTAGGTG
nad2 a F	GGATCCTCCCACACATGTTC
nad2 a R	GCGAGCAGAAGCAAGGTTAT
nad5 a F	TGGACCAAGCTACTTATGGATG
nad5 a R	CCATGGATCTCATCGGAAAT
rpl2 F	CCGAAGACGGATCAAGGTAA
rpl2 R	CGCAATTCATCACCATTTTG
rps4 F	ACCCATCACAGAGATGCACA
rps4 R	TCACACAAACCCTTCGATGA
**Circular RT-PCR**	
atp1 RT-407R	GGGGCTTTCACTTCGACACGTC
atp1 RT5BR	TTTAAGGGCTTGTAGTAATTCAGGTTT
atp1 cRT-195R	GGCCATTCCTTTCACACCGTTGG
*atp1 c*RT5DR	TTCAGGTTTRACACTATTTRGAATGGC
atp1 cRT2F	GGTGGATTAACTAACGAAAGAAAAATGGAAC
atp1 cRT3F	GAGGTGCAAGGCTGACAGAAGTAC
**Biotinylated primers for Northern blots**	
404 AS	GCTTTCACTTCGACACGTCTTT
1457 AS	TCCACCTTTAAGGGCTTGTAGT

Supplemental Table S11. Antibodies used in this work.

	dilution	Reference
Nad9	1/50,000	Lamattina et al (1993) FEBS, **217**(3), p 831-8
RISP	1/5,000	Carrie et al (2010) J Biol Chem 285 (46), p 36138-48
Cox2	1/5,000	Agrisera AS04 053A
Atp1	1/1,000	Tom Elthon, University of Nebraska
HSP70	1/2,000	Tom Elthon, University of Nebraska
AOX	1/1,000	Elthon et al (1989) Plant Physiol 89 (4), p1311-7
Porin	1/5,000	Tom Elthon, University of Nebraska
FLAG	1/1000	Sigma F3165

SUPPLEMENTAL MATERIALS AND METHODS

Trait Analysis

In T3 generation, three replicates of each genotype were grown for phenotypic investigation. Rosette diameter was measured, and leaf numbers were counted in week 2 and week 4, respectively. Plant height was measured in week 4 and week 6. Bolting and flowering times from germination were recorded. Silique length, silique number per plant and seed weight were measured at maturation stage.

The root lengths of 10-day-old seedlings were measured using the Fiji ImageJ

software on 5 plates containing about 40 seedlings each for native RPF2, RPF2-*atp1*-16 and RPF2-*atp1*-9 lines in T4 generation. The seedlings were grown on vertical plates under a 16-hour photoperiod. Primary root tips were identified and marked manually in the images; root lengths were estimated as the linear distance between the root tip and the base line where the seeds were placed. The dashed lines show the average root length for each genotype

Respiration Measurements

Two groups of T3 generation plants were grown under long-day conditions (16h/8h; light/dark) and short-day conditions (8h/16h; light/dark) for four weeks in two separate growth cabinets. Twenty-four hours prior to respiratory measurements, plants with mature leaves in the range of 60 – 110 mg were placed in pre-designated spots in a growth cabinet to ensure that each pot received similar intensity of light (120 - 140 photons µmol $m^{-2}s^{-1}$). Respiration measurements were carried out using a Q2 O_2-sensor (Astec Global, Maarssen, The Netherlands). Harvested mature leaves were placed in 2 ml tubes and hermetically sealed with specialized caps (Astec Global). The frequency of oxygen concentration measurements was set to three minutes. The slope of O_2 consumption (R_N rate) was then calculated between three and five hours after the start of the run, which is 10 – 13 hours into the plant's dark period. Molar O_2 consumption was calculated using the ideal gas law and the O_2 partial pressure was determined to be 20.95% of the atmospheric pressure [O'Leary, 2017 #167; Scafaro, 2017 #64]. The calculated R_N rate for individual leaves were normalized to their corresponding fresh weights. Statistical analyses were performed using XLSTAT (ADDINSOFT, Paris, France) where indicated. One-way analysis of variances (ANOVA) was first conducted to compare R_N rates between genotypes. Significant differences observed in the one-way ANOVA was then further analysed using Tukey's HSD test.

Absolute quantitation of AMP, ADP and ATP by LC-MS

Approximately 25 mg leaves or roots of 2-week-old seedlings grown vertically on plates, or 20 µg isolated mitochondria were collected and immediately snap-frozen in liquid nitrogen. For seedlings, samples were then ground to fine powder. Ice-cold 15% TCA solution (1 ml) was added to samples, supplemented with $^{13}C_5$,$^{15}N_5$-AMP as an internal standard. Following centrifugation at 24,000 × *g* for 10 min (4°C), 1 mL 78/22 dichloromethane/trioctylamine was added to the supernatant. The mixture was then vortexed and centrifuged at 5,000 × g for 2 min. The upper phase was collected and diluted in 1 mL H_2O and 5 µL 0.5% acetic acid. The resulting mixture was applied to a Strata X-AW SPE cartridge (pre-equilibrated with 1 mL methanol, 1 mL 2/25/73 formic acid/methanol/H_2O, and 1 mL 10 mM ammonium acetate pH 4.5) and the flow-through was discarded. The cartridge was then washed with 1 mL of 1 mM ammonium acetate (pH 4.5) and 1 mL methanol before nucleotides were eluted with 0.5 mL 20/80 ammonia/methanol twice. The eluate was transferred to a new tube and dried using a SpeedVac.

Dried samples were resuspended in 100 µL 5-mM ammonium acetate (pH9.5)/acetonitrile (95/5). chromatographic separation was performed using Agilent Poroshell 120 HILIC-Z column, using mobile phases of 5-mM ammonium acetate (pH9.5)/acetonitrile (90/10) (solvent A) and acetonitrile (Solvent B). The elution gradient was 100% B at 0 min, 60% at 5 min, 30% at 5.5 min, 30% at 7 min, 15% at 7.5 min, 15% at 8.5 min, 100% at 9 min and 100% at 22 min. The column flow rate was 0.3 mL/min; the column temperature was 35 °C, and the autosampler was kept at 10°C. Data acquisition and LC-MS control were carried out using the Agilent MassHunter Data Acquisition software (version B06.00 Build 6.0.6025.4). The autosampler was kept at 10°C. The QQQ-MS was operated in SRM mode in positive ion polarity using the following settings: capillary voltage, 3000V; drying N2 gas and temperature, 12

L/min and 250 °C respectively; Nebulizer, 15 psi. All optimised SRM transitions for each target were listed in the Table below. Data analysis was carried out using MassHunter Quantitative Analysis Software (version 10.1, Build 10.1.733.0). Metabolites were quantified by comparing the integrated peak area with a calibration curve obtained using authentic standards and normalised against fresh

Optimised SRM Transitions for the Adenylate Measurements

Compound	Precursor ion (m/z)	Product ion (m/z)	RT (min)	Collision energy (mV)
$^{13}C_5$-$^{15}N_5$-AMP	363	146	7.4	16
$^{13}C_5$-$^{15}N_5$-AMP	363	101.9	7.4	32
ADP	428	348	7.55	16
ADP	428	136	7.55	24
ATP	507.9	410	7.7	16
ATP	507.9	348	7.7	16
ATP	507.9	136	7.7	36

Proteomics Analysis

For quantitative untargeted mass spectrometry, crude mitochondrial pellets were obtained from 3-week-old WT, RPF2 native, RPF2-*atp1-9* and RPF2-*atp1-16* seedlings grown on plates and the samples from 3 independent experiments were prepared as previously described (Colas des Francs-Small *et al.*, 2014; Petereit et al., 2020).

Two hundred µg of protein were precipitated with 9 volumes cold acetone for 24h at −20°C, resuspended in 100 µl 50 mM ammonium bicarbonate, 10 mM dithiothreitol (pH 8.0), and incubated at 58°C for 20 mins. Samples were cooled to room temperature and alkylated with 100 µl 50 mM ammonium bicarbonate, 25 mM iodoacetamide for 20 minutes in the dark. The samples were digested in 1:50 (w/w, trypsin/protein) trypsin, 50 mM ammonium bicarbonate, 2% (v/v) acetonitrile, 1.2 mM $CaCl_2$, 0.1 M guanidine HCl, pH 8.0) at 37°C for 16 hours under agitation (1000 rpm). Digested samples were desalted and concentrated using C18 macroSpin columns (The Nest Group, USA) and eluted with 100 µL of 80% acetonitrile, 0.1% formic acid solution. Eluates were dried under vacuum, resuspended in 2% (v/v) acetonitrile, 0.1% (v/v) formic acid to a final concentration of ~1 $\mu g \cdot \mu L^{-1}$ protein, and filtered through Ultrafree-MC 0.22 µm, PVDF Centrifugal Filters (Millipore Sigma, USA).

Samples were analysed by LC-MS on a Thermo Exploris 480 mass spectrometer using data-dependent acquisition. Analysis consisted of direct injection onto a self-packed 150 mm x 75 µm Dr Maisch Reprosil-Pur 120 C18-AQ 1.9 µm column. Water/acetonitrile gradients with 0.1% formic acid were formed by an Ultimate U3000 nano pump running at 250 $nL \cdot min^{-1}$ from 2-30 % acetonitrile over 76 minutes.

Thermo raw files were database searched and quantified using MaxQuant (v1.6.10.0) [Cox, 2008 #176]. Sixteen samples were analysed in all (4 from RPF2-*atp1-9* plants, 4 from RPF2-*atp1-16* plants, 4 from WT plants and 4 from WT plants expressing WT RPF2). The data were filtered to remove proteins lacking intensity values from more than a quarter of the samples, leaving 410 proteins for analysis. The remaining missing values were imputed using singular value decomposition (https://github.com/invenia/Impute.jl), normalised (using the same approach as DESeq2) and then used to estimate phenotype-dependent factors and p-values for differential abundance for each protein using a linear model. P-values were adjusted for multiple testing using the Benjamini-Hochberg procedure.

RNA-seq Data Analysis

For analysis of differential nuclear gene expression, reads were assigned to transcripts from the *Arabidopsis* transcriptome (Araport11) with Salmon v0.11.2 and the count data analysed with DESeq2 v1.16.1 (Love et al., 2014). GO term enrichment analysis was carried out with the topGO package.v 2.28.0 (Alexa et al., 2006) using GO terms assigned by TAIR (org.At.tair.db from BioConductor release

3.15). The control groups for the GO term enrichments were chosen such that their mean expression level matched that of the differentially expressed genes (using the genefinder function from the BioConductor genefilter library and a Manhattan distance of 10). For analysis of mitochondrial gene expression, reads were mapped to the mitochondrial genome (accession BK010421) with BBMap (https://sourceforge.net/projects/bbmap/). Read coverage was quantified with Pyrimid (https://github.com/ian-small/pyrimid) (the same nucleotide counts were used for analysis of RNA editing), normalised for library size using the same method as DESeq2, normalised for coding sequence length (summing counts and lengths for each exon) and finally expressed as transcripts per thousand mitochondrial transcripts. For analysis of splicing, reads mapping to donor and acceptor splice junctions (as annotated in BK010421) were checked for kmers specific to unspliced or spliced transcripts and counted accordingly. For analysis of RNA editing, nucleotide counts from Pyrimid (https://github.com/ian-small/pyrimid) were analysed using a binomial test to identify sites likely ($p < 0.05$) to be edited in more than 5% of transcripts. Of the 510 sites detected, the 410 that were in coding sequences were retained for calculation of odds ratios and visualisation. Odds were calculated as (1 + edited count)/(1 + unedited count).

For analysis of potential cleavage sites, 5' ends of read 2 data were taken as potential 5' RNA termini as this marks the position of the ligation of the 5' adapter in the Illumina TruSeq Stranded library preparation protocol. These 5' ends represent a mixture of original 5' termini in the RNA sample plus those generated by RNA fragmentation prior to ligation of the adapter. The frequency of 5' ends at each genome position was smoothed and normalised to the local background (foreground mean / background mean, where the foreground window was 10 nt and background window 2000 nt). For statistical analysis, the mean smoothed, normalised counts for RPF2-*atp1* samples were converted to peaks (Fink and Pratt, 2018) and troughs and the peaks filtered by prominence (>4); for each prominent peak, the maximum counts within the peak for all samples were compared using a one-tailed t-test (looking for higher peaks in RPF2-*atp1*) and the *p*-values adjusted for multiple testing (Šidák correction). For the reverse strand transcripts, 364882 nucleotide positions were compressed into 5371 peaks of which 77 had a prominence >4; two of these, centred at 67233 (*atp1*) and 98564 (*nad2*) were significant by the test used.
