# Supplementary material for: Mitochondrial atp1 mRNA knockdown by a custom-designed pentatricopeptide repeat protein alters ATP synthase
Source: Plant Physiol. 2024 Jan 11;194(4):2631–47. doi: 10.1093/plphys/kiae008 (PMC10980415; doi:10.1093/plphys/kiae008)
Supplement: kiae008_Supplementary_Data [file pp.01300.2023-s06.pdf › pp.01300.2023-s06.pdf]

Plant Physiology®

Knockdown of mitochondrial atp1 mRNA by a custom-designed pentatricopeptide repeat protein alters F1Fo ATP synthase

Fei Yang, Lilian Vincis Pereira Sanglard, Chun-Pong Lee, Elke Stroher, Swati Singh, Glenda Oh, A. Harvey Millar, Ian Small, and Catherine Colas des Francs-Small

Review Timeline:

Submission Date:	08-Nov-2022
Editorial Decision:	20-Dec-2022
Resubmission Received:	30-Aug-2023
Editorial Decision:	14-Nov-2023
Revision Received:	28-Nov-2023
Accepted:	30-Nov-2023

20-Dec-2022

Dr. Catherine Colas des Francs-Small
The University of Western Australia
ARC Centre of Excellence Plant Energy Biology
Perth
Australia

RE: Knockdown of mitochondrial atp1 mRNA by a custom-designed pentatricopeptide repeat protein alters F1Fo ATP synthase

Dear Dr. Colas des Francs-Small,

Thank you for giving us the opportunity to review your manuscript for publication in the Plant Physiology. As these concerns are of some gravity and will entail substantial changes to the manuscript, you are likely to need additional time to address them. I must therefore reject the manuscript in its present form. Nonetheless, if you are able to address the points raised in review, I would welcome your resubmission of a manuscript that incorporates your responses.

The reviewers agreed that your manuscript reports interesting work on manipulating mitochondrial atp1 expression, both in terms of the novelty of the method and the biochemical/physiological consequences. However, they felt that it would benefit from a more rigorous presentation and possibly additional data to strengthen your conclusions. In particular, I direct your attention to two points for consideration in a resubmitted manuscript. Firstly, Reviewer 1 had problems with accessing some of the 'omics data. Secondly, the need for ATP and respiration measurements in leaves was suggested and this might require further experimental data (or possibly refocussing the manuscript structure/conclusions. Please also address all the other points raised by the reviewers.

Please note that any resubmission will be treated as a new entry to the system and is likely to go out for review.

If you do have any queries or concerns, you are welcome to contact me in the first instance.

Thank you again for allowing Plant Physiology to review your work. We look forward to hearing from you soon.

Sincerely,

Plant Physiology Board of Editors

-- Reviewer comments:

Reviewer #1 (Comments for the Author):

In this manuscript, Yang et al. report about the engineering of a synthetic PPR protein, derived from RPF2, targeting the Atp1 mitochondrial RNA. This synthetic PPR can cleave the Atp1 transcript (leading to a ca. 5-fold decrease), which subsequently strongly affects the assembly of ATP synthase. This in turn impairs the growth of the transgenic plants. The authors also show that the RPF2-atp1 construct leads to higher respiratory levels, and they combined several omics approaches to determine the metabolic adjustments, which lead to this reduced growth.
The manuscript is well written; even though a few sentences remain convoluted and will require editing.
In its current form, this work does not provide full satisfaction, mostly due to the second part of the study. Indeed, through PPR engineering, the manuscript offers a valuable method to modulate mitochondrial (and potentially plastidial) transcripts, one of the holy grails for research groups working with organelle genes editing. A few additional controls (including gel shifts etc) would still be required (as listed below in part C). However, the second part of the study, which integrates -omics, lacks depth, and appears way too superficial in many aspects, ranging from experimental design to the interpretation of the results. The arguments supporting this point of view are provided here below under 3 main topics (A-C):

A -There is a problem with the original data provided by the authors. There are either not accessible, or clearly absent as described below:

1) The proteomic data cannot be accessed.
From the website, http://proteomecentral.proteomexchange.org/cgi/GetDataset?ID=PXD037659
one gets the following info:
If you are a reviewer of a manuscript that includes this dataset:

• You should also have received a username and password to access this dataset PXD037659. (yes, it was provided by the authors)

• If you did, go to the PRIDE Archive login page https://www.ebi.ac.uk/pride/archive/login to enter the reviewer credentials and access the data. (the user is sent to an 404 error page)
Unfortunately, while the authors have provided the credentials, one cannot access the data as the link in the second "bullet point", refers to a 404 error page. Clearly not the fault of the authors, but data are not accessible (at least a few times when I tried).

2) The raw data for metabolomics are not presented. These must be provided, including replicates and statistical analysis. Statistics should also be added to the figure legends.

3) For RNAseq, only raw data files are accessible at the NCBI. A detailed analysis should also be provided. Moreover, I am a bit surprised by the number of targets identified by RNAseq, a bit less than 19000 in the supp. table provided. Any reasons why? Also, the supp table is very "raw", with the description being quite unreadable. Please edit to present something a bit more user-friendly, or at least, "reader-friendly".

4) The analysis for GOs is very preliminary, and should be provided. The authors have only provided a sentence in the supp. experimental procedure file. "GO term enrichment analysis was carried out with the topGO package.v2.28.0 (Alexa et al., 2006)." This is far from satisfying. Also, Figure 4 as it stands now, is not satisfactory. One needs to know the genes contributing to each term, the size of the terms, the references used, the potential enrichments. Why were only Biological Process terms used? A log of the pvalue was used; why not, but at least, a significance threshold for the reader should be displayed. A proper, detailed, analysis is required.

B - The growth conditions used for the experimental setup are very variable. Indeed, there is a high heterogeneity in the growth conditions and biological material sampled throughout the study. This may be problematic for the interpretation of the physiological data. Furthermore, additional info is required, such as what part of the samples were harvested and when; under which growth conditions. Apparently, some seedlings were grown on plates, while other ones in liquid medium perhaps (for metabolomics?). This can make a big difference. RNAseq were performed with 6W old leaves and flower buds: I suppose it comes from plants grown in long days, but were all rosette leaves selected? Or only a few? If so, which ones? Etc... This truly lacks rigour.

To illustrate the point: growth details extracted from the M&M section

- Total RNA extraction and Northern Blotting
Total RNA was extracted from 4-week-old rosette leaves using PureZol kits (Bio-Rad).

- RT-qPCR
Total RNA was isolated from 2-week-old seedlings grown on plates under 16-hour photoperiod

- RNA Sequencing
Total RNA was isolated from 6-week-old rosette leaves and bolting flower buds of Col-0 and one of the RPF2-atp1 (RPF2-atp1-9) transformed lines (T3)

- Proteomics Analysis
For quantitative untargeted mass spectrometry, crude mitochondrial pellets were obtained from 3-week-old WT, RPF2 native, RPF2-atp1-9 and RPF2-atp1-16 seedlings grown on plates

- For the ATP synthesis rate, 20 µg of purified mitochondria isolated from 3-week-old native RPF2, RPF2 atp1-9 and RPF2 atp1-16 seedlings

- Mass Spectrometry Analyses of Organic Acids and Amino Acids
Four-week-old seedlings (~25 mg) grown under a 16-h light/8-h dark photoperiod were collected at the end of the night and snap-frozen in liquid nitrogen

C - A list of points to address/consider:
• 42: "cytoplasmic male sterility (CMS)" does not appear as a relevant keyword for the work described here. Please remove.

• 51: "... as the lack of reliable methods to transform mitochondria ...". This statement should be tempered as the use of the TALEN technology (mentioned after in the manuscript) looks quite effective (e.g. Forner et al., 2022).

• 122: "... (Dahan and Mireau, 2013; Gaborieau et al., 2016; Wang et al., 2021)]". Please, remove "]"

• 150: Figure 1A: Native RPF2 biomass looks higher than WT. Also, does the native RPF2 contains the native targeting sequence or the FDH transit peptide? Is it possible to get the construct with FDH transit peptide + native RPF2 + FLAG tag to

assess the impact of the Tag in C-ter and transit peptide?

• 181: a gel shift showing the actual interaction between RPF2-atp1 and Atp1 is necessary.

• 216: "RPF2-atp1-2 plants showed some instability across generations ...". That is a really important point for biotechnology applications. How stable are these transgenic lines?

• 245: "... with their reduced fertility". Could the authors provide an Alexander staining to support this claim?

• 256-271: As it stands, this section does not add much information. It should be developed. What is the impact the ANAC017-dependent/independent transcripts in this context?
• 287: "... This second peak does not represent an RNA cleavage site ...". Again, a gel shift showing a potential interaction between RPF2-atp1 and Nad2 is necessary.

• 292: "Apparent relative editing rates in WT as compared to RPF2-atp1-9 were also calculated for mitochondrial editing sites within coding sequences and found to be generally lower in the modified plants ...". Seeing the phenotype of RPF2-atp1 plants, it is not so surprising. Stress is likely to affect RNA editing. Could the authors discuss the impact, if any, of such difference?

• 356-360 (+ Supp Table 8): "...detailed analysis..." there is nothing detailed here. This list selects only 36 genes associated with amino acid synthesis and degradation pathways out of 377. Why only these ones? Please provide an explanation.
• 433: "... (Van Aken et al., 2009; Schwarzlander et al., 2012; De Clercq et al., 2013) ". Please edit to SchwarzlÄnder.

• 531: "... RPF2-atp1 gene with a 3xFLAG ...". Please, mention that in RPF2-atp1 the 22 first aa are missing (presumably the transit peptide?). And if so, why did the authors not use RPF2 native transit peptide as RPF2 is supposed to be targeted to mitochondria?

• 354: "The latter represents 20-30% of the total amino acid pool as Ser family amino acids in RPF2-atp1 compared to only 13% in WT". A bit confusing, please rephrase.

• 408: "The conversion of Gly to Ser is a major source of NADH in leaf mitochondria and puts high demands on the respiratory chain as an electron sink (Krömer, 1995) (...)". Well, in leaves, this can be buffered by the malate valve since MDH provides NAD+ for GDC, and thus export of makate would alleviate pressure on the respiratory chain. I do not think that stating "puts high demand on the respiratory chain" is still the way of seeing this redox echange.

• 457-485: This entire paragraph does not make much sense to me. The authors performed a metabolomic analysis on seedlings at the end of the night, and they discussed a typical gly/ser ratio under photosynthetic conditions i.e. under light and therefore high photorespiratory flux. Such flux does not exist at the end of the night... What likely happens in these plants, is that there is a lack of energy to support the carbon assimilation, hence the plants accumulate amino acids as a safe source for nitrogen storage. The malate/fumarate imbalance depicted at the TCA cycle is probably incorrect. It is more likely reflecting the accumulation of photoassimilates during the day, as cytosolic/vacuolar fumarate in the WT, as compared to the mutant that is limited for redox exchange and would therefore have a lower level of fumarate throughout day and night.

• In line with the comments above, Figures 4, 5, 6 and 7 are a bit superficial, and would require additional work to be convincing, and provide more depth to the reported study.

To summarize, this work is interesting and has a good potential, but currently it falls a bit short due to a lack of rigour in the -omics analyses. It requires additional efforts to be fully convincing.

Reviewer #2 (Comments for the Author):

First of all, I truly apologize that it took longer than usual to assess the manuscript due to other commitments.

The manuscript by Yang and colleagues is an interesting attempt to enhance our knowledge about the importance of the mitochondrial F1Fo ATP synthase in Arabidopsis thaliana by using custom-designed PPR proteins. The topic itself is already a very complex process because mitochondrial transformation methods are rather complicated and only a few labs around the world use it successfully. The group of researchers around Cath Francs-Small have already successfully induced changes in mitochondrial transcript levels by using a custom-designed PPR protein. Here, The authors have specifically investigated how knockdown atp1 transcripts impacted growth and fertility in Arabidopsis.
In the first part of the manuscript, the authors generated a range of mutants and following a well-planned and performed screening out of 32 independent transformants carrying the RPF2-atp1 construct and observed that 14 displayed slow growth and delayed flowering. They further confirmed that high expression of RPF2-atp1 lead to a reduced atp1 transcript accumulation. By further performing BN-PAGE on a crude extract from leaf membranes the authors observed significant

changes in F1Fo ATP synthase in transformant plants. They further showed that mitochondrial-related proteins are also modified in these transformants. Together, such changes likely lead to a reduction in both shoot and root despite an increased oxygen consumption. Further RNAseq analysis confirmed that reproductive development is indeed affected yet no mitochondrial off-target was observed. Further untargeted proteomics revealed that F1 assembly was significantly impacted. Analysis of root adenylates pools revealed a slower ATP synthesis rate yet the adenylate pool is maintained in vivo. Changes in amino acids and organic acids were also evident in the transformant lines.
The manuscript is well written, the experiments are well conducted and the data is adequately analysed. The results are really interesting, and the reasoning is mostly fine, but the presentation could be improved. Altogether this is a nice piece of work but the objectives of the study are not well defined and I only have a few points that should be explained in detail.

The authors should also pay attention to a few points below:

1) It must be clearly defined (and perhaps justified) the plant age of each analysis. Since growth parameters were obtained in 4-week-old plants it is rather strange that other analyses were performed in plants with different ages (meaning, different developmental stages) without clear justification. My feeling is that coupled with day length may explain some of the changes observed (see lines 203-231).
2) Lines 321-325:Although the authors attempted to explain why they used root tissues to analyse adenylate pools, I think it is rather complicated to connect this only measured parameter with all other data obtained in leaf tissues. I strongly recommend the authors to measure adenylate pools in leaf tissues.
3) Line 225: What the authors are trying to state with "To minimize developmental stage differences between genotypes, short day conditions (8/16 hours, day/night) were applied"? This is the first time that day length is mentioned and it makes the reader wonder what are the reasons behind it.
4) Result for the RNAseq data lines 233-294. Because huge changes in transcriptomes were detected, the authors extensively described such changes in transcriptomes. However, such descriptions of the changes in transcriptomes are speculative and don't reinforce the authors' main conclusions. For the key genes discussed in the transcriptome section, please confirm the expression by other experiments to validate functionally, rather than simply discussing RNAseq data only.
5) It is also important to remember that metabolite profiling here obtained by LCMS gives steady-state values and changes in those must be interpreted with caution. An increased steady-state level is usually interpreted as a block in the metabolic pathway immediately after the intermediate, in other words as reduced consumption of that intermediate. However, it could also the caused by an increased synthesis of that intermediate or by both.
6) I also wonder whether the development phenotype was maternally related. Additional experiments or text must be added to confirm that only maternal tissue contributed to the atp1 phenotype. However, the authors have only taken a snapshot picture of the seed development phenotype, and I also wonder whether this phenotype is observed since the flower appearance as well as with the number of seeds per silique.
7) What is the seed germination phenotype in atp1 transformants? Does it only apply in a maternal fashion? If there is a causative link between developmental phenotype and germination rate then there would need to be a maternal link to maintain the pattern.
8) Throughout the main text: is development in the atp1 mutants compromised/reduced or just delayed? This could be important for trying to understand the nature of the phenotype.
9) The discussion is a bit frustrating and circular mainly because it is lengthy and suffers from over-interpretation. There are some points in the discussion that have been previously commented on in the introduction. I suggest avoiding the duplicity of information.
10) Because of the many indirect pieces of evidence, it is sometimes not easy to follow the idea or the conclusion. I guess that particularly non-experts in this field might have problems following the interpretation of the results. It might help to present the more direct shreds of evidence in the form of a schematic model summarizing the main results and connecting it with the phenotype observed.

Minor comments

1) The authors are somehow mixing reducing seed yield (Supplemental Figures 3 and 4) and reduced fertility (line 245). The authors have not determined seed germination or pollen viability so they should keep the focus on seed yield.
2) The discussion is circular in places and frustrating, particularly at the beginning where there is a clear repetition of results and methods already described. Thus, the discussion should be reduced in length to give a short overview of the results and emphasize the important integrative concepts and explain how this work informs future thinking.
3) Minor yet rather important point: It is rather strange and disappointing to see that several seminal papers/reviews from the groups around the German groups (Braun, Binder, Fernie, Maurino, Schwarzlander) working with plant mitochondria proteome and metabolism (including amino acids and organic acids) have been totally forgotten in this manuscript. I am sure the authors are aware of such work.

Reviewer #3 (Comments for the Author):

The authors describe the isolation and characterization of mutants defective for the Atp1 subunit of mitochondrial ATP synthase of A. thaliana. The method used to generate the mutants relies on the expression of a pentatricopeptide repeat (PPR) protein

designed to cleave the atp1 mRNA. This method is original and if generasible, could represent an efficient way to alter mitochondrial gene expression in land plants.

The description of the mutants is well detailed. This description also raises some questions which are not yet fully answered/discussed in the manuscript.

1.The mutants show increased respiration rate. Which explanation could be provided? Which pathway (cytochrome or alternative) is responsible for the increased rate? Adding inhibitors specific to the alternative pathway (SHAM) or the cytochrome pathway (KCN) could be useful, especially considering the increased abundance of AOX.

2. The involvement of the phb3 and phb3 anac017 double mutants should be better introduced in the RNA seq results. Comparison of RNA seq data from other mutants such as those affected in respiratory complexes could also be useful.

3. The fact that the ATP, ADP and AMP levels are close to the WT in the mutants is quite intriguing and the analyses (metabolomics, proteomics) performed do not allow to understand this point. ATP production in the chloroplasts could be increased which would explain the relatively good growth of the mutants. Why not measure the ATP content also in the leaves? Photosynthetic measurements may also help, considering the crosstalk between chloroplasts and mitochondria. This point could at least be discussed.

4. Discussion line 392-393: are there examples where the biogenesis of one respiratory complex is dependent on the synthesis of others?

Responses to the reviewers, manuscript PP2022-RA-01591 :
Reviewer #1:

A -There is a problem with the original data provided by the authors. There are either not accessible, or clearly absent as described below:

1) The proteomic data cannot be accessed.
From the website, http://proteomecentral.proteomexchange.org/cgi/GetDataset?ID=PXD037659 one gets the following info:
If you are a reviewer of a manuscript that includes this dataset:
• You should also have received a username and password to access this dataset PXD037659. (yes, it was provided by the authors)
• If you did, go to the PRIDE Archive login page https://www.ebi.ac.uk/pride/archive/login to enter the reviewer credentials and access the data. (the user is sent to an 404 error page)
Unfortunately, while the authors have provided the credentials, one cannot access the data as the link in the second "bullet point", refers to a 404 error page. Clearly not the fault of the authors, but data are not accessible (at least a few times when I tried).

Response: This issue seems to be resolved as far as I can tell. I have no issues accessing with the reviewers' login credentials provided.

2) The raw data for metabolomics are not presented. These must be provided, including replicates and statistical analysis. Statistics should also be added to the figure legends.
Response: The data are presented with the statistical analyses in the Excel files supplied as Supplemental Table 5.

3) For RNAseq, only raw data files are accessible at the NCBI. A detailed analysis should also be provided. Moreover, I am a bit surprised by the number of targets identified by RNAseq, a bit less than 19000 in the supp. table provided. Any reasons why?
Also, the supp table is very "raw", with the description being quite unreadable. Please edit to present something a bit more user- friendly, or at least, "reader-friendly".

Response: The raw RNA-seq data (sequence reads) are available from the NCBI, all the gene expression data is in Table S2, and our interpretation of the data is in the manuscript. It is not clear what else the reviewer wants to see. To see ~19000 transcripts detected by RNA-seq seems to us normal and expected; is the reviewer under the misapprehension that only differentially expressed transcripts are listed in Table S2?
As requested, Supplemental Table 2 (Table S2) has been simplified and the information displayed in individual columns, making it easier to visually browse.

4) The analysis for GOs is very preliminary, and should be provided. The authors have only provided a sentence in the supp. experimental procedure file. "GO term enrichment analysis was carried out with the topGO package.v2.28.0 (Alexa et al., 2006)." This is far from satisfying. Also, Figure 4 as it stands now, is not satisfactory. One needs to know the genes contributing to each term, the size of the terms, the references used, the potential

enrichments. Why were only Biological Process terms used? A log of the pvalue was used; why not, but at least, a significance threshold for the reader should be displayed. A proper, detailed, analysis is required.

Response: More details for the GO term analysis have been added in the Supplemental Experimental Procedures section. Biological Process terms were used because it was biological processes we were most interested in. In Figure 4, all points are highly significant and the significance threshold would be barely distinguishable from the x-axis, as 1 = log (0.1). A scale showing the number of genes for various disc areas is now provided. The information requested by the reviewer is (and was) provided in Tables S3 and S4. The lists of genes in each category could be added if necessary, but we fear represent a huge amount of unnecessary data.

B - The growth conditions used for the experimental setup are very variable. Indeed, there is a high heterogeneity in the growth conditions and biological material sampled throughout the study. This may be problematic for the interpretation of the physiological data. Furthermore, additional info is required, such as what part of the samples were harvested and when; under which growth conditions. Apparently, some seedlings were grown on plates, while other ones in liquid medium perhaps (for metabolomics?). This can make a big difference. RNAseq were performed with 6W old leaves and flower buds: I suppose it comes from plants grown in long days, but were all rosette leaves selected? Or only a few? If so, which ones? Etc... This truly lacks rigour.

Response: All through the results section, we detailed what parts of the plants were harvested, and explained the choice of the stage and the growth conditions for the various experiments. None of the plants were grown in liquid medium. They were grown on plates under long day conditions (16h light/8 hours light), and the seedlings were harvested after 3 weeks at the end of the dark period for metabolomics studies and initial nucleotide measurements, as well as mitochondrial enrichment (proteomics) or purification (mitochondrial ATP synthesis measurements). For phenotypic observations and for RNA-seq analysis, the plants were germinated on plates and transferred to soil. For RNA-seq, only the central leaves of the rosette and the emerging buds of the bolting plants (actively dividing tissues, developing buds enriched in mitochondrial transcripts) were harvested.

C - A list of points to address/consider:
• 42: "cytoplasmic male sterility (CMS)" does not appear as a relevant keyword for the work described here. Please remove.

Response: "cytoplasmic male sterility (CMS)" has been removed from the key words

• 51: "... as the lack of reliable methods to transform mitochondria ...". This statement should be tempered as the use of the TALEN technology (mentioned after in the manuscript) looks quite effective (e.g. Forner et al., 2022).

Response: In this paper, the authors performed targeted mutation of the mitochondrial genome, but not transformation. For this reason, the statement line 51 has not been

modified, but the two recent papers using this technology have been added to the introduction (line 110).

• 122: "... (Dahan and Mireau, 2013; Gaborieau et al., 2016; Wang et al., 2021)]". Please, remove "]"

Response: the extra bracket has been removed.

• 150: Figure 1A: Native RPF2 biomass looks higher than WT. Also, does the native RPF2 contains the native targeting sequence or the FDH transit peptide?
Is it possible to get the construct with FDH transit peptide + native RPF2 + FLAG tag to assess the impact of the Tag in C-ter and transit peptide?

Response: The native construct has the FDH transit peptide like the RPF2-*atp1* construct, but an N-terminal FLAG tag. We did not generate this construct with the C-terminal tag, but it is not expected to affect plant growth.

• 181: a gel shift showing the actual interaction between RPF2-atp1 and Atp1 is necessary.

Response: As opposed to synthetic PPR proteins, native PPR proteins are very prone to aggregation. Despite several attempts, we were not able to obtain monomeric RPF2-*atp1* recombinant protein by size exclusion. We therefore are unable to present conclusive gel shift assays due to protein aggregation. The fact that RPF2-*atp1* is active *in vivo* and able to recruit the endonuclease that cleaves the *atp1* transcript downstream of the predicted site nevertheless proves that it binds its target *in vivo*. This is commented in the discussion lines 519-522.

• 216: "RPF2-atp1-2 plants showed some instability across generations ...". That is a really important point for biotechnology applications. How stable are these transgenic lines?

Response: Apart from RPF2-*atp1-2*, the other lines presented in the manuscript have been stable through many generations (6 or 7).

• 245: "... with their reduced fertility". Could the authors provide an Alexander staining to support this claim?

Response: This claim has been removed from the text.

• 256-271: As it stands, this section does not add much information. It should be developed. What is the impact the ANAC017-dependent/independent transcripts in this context?

Response: We believe this is interesting information to follow-up in the future but goes beyond the topic of this paper.

• 287: "... This second peak does not represent an RNA cleavage site ...". Again, a gel shift showing a potential interaction between RPF2-atp1 and Nad2 is necessary.

Response: For reasons explained above, we are unable to present gel-shift assays, but this peak represents an intron end and not a new cleavage site, and therefore we do not expect RPF2-*atp1* to bind there.

• 292: "Apparent relative editing rates in WT as compared to RPF2-atp1-9 were also calculated for mitochondrial editing sites within coding sequences and found to be generally lower in the modified plants ...". Seeing the phenotype of RPF2-atp1 plants, it is not so surprising. Stress is likely to affect RNA editing. Could the authors discuss the impact, if any, of such difference? "

Response: We commented on this in the discussion, lines 439–443: "The observation that splicing and editing rates of mitochondrial transcripts were reduced in the modified plants is a reminder that RNA processing is sensitive to the physiological state of the plants and thus defects in splicing and editing in mutants are not always indicative of a direct role of the missing protein in these processes."

• 356-360 (+ Supp Table 8): "...detailed analysis..." there is nothing detailed here. This list selects only 36 genes associated with amino acid synthesis and degradation pathways out of 377. Why only these ones? Please provide an explanation.

Response: only a set of genes with significantly differential expression and associated with amino acid synthesis and degradation was presented in Supplemental Table S8. The new Table S9 now contains all the genes from the 377 that are present in our RNA-seq data.

• 433: "... (Van Aken et al., 2009; Schwarzlander et al., 2012; De Clercq et al., 2013) ". Please edit to SchwarzlÄnder.

Response: Corrected

• 531: "... RPF2-atp1 gene with a 3xFLAG ...". Please, mention that in RPF2-atp1 the 22 first aa are missing (presumably the transit peptide?). And if so, why did the authors not use RPF2 native transit peptide as RPF2 is supposed to be targeted to mitochondria?

Response: The first 22 amino acids from RPF2 are part of the predicted mitochondrial targeting sequence (comprising 39 amino acids) which was replaced with that of FDH in the RPF2-atp1 construct, as previously described for the RPF2 and RPF2-*nad6* constructs (Colas des Francs-Small et al, 2018). The FDH targeting sequence provides a convenient tool to distinguish modified vs native RPF2 by PCR. Part of the predicted targeting sequence was kept to ensure that no residue potentially important for the proper folding at the N-terminal end of the protein would be missing. Indeed, our results show that the modified protein is correctly addressed to the mitochondrion.

• 354: "The latter represents 20-30% of the total amino acid pool as Ser family amino acids in RPF2-atp1 compared to only 13% in WT". A bit confusing, please rephrase.

Response: The text was changed to "This indicates that 20-30% of the total amino acid

content in the samples are Ser family amino acids in RPF2-*atp1* while these amino acids only represent 13% of the total amino acid content in WT" (lines 364-366).

• 468: "The conversion of Gly to Ser is a major source of NADH in leaf mitochondria and puts high demands on the respiratory chain as an electron sink (Krömer, 1995) (...)". Well, in leaves, this can be buffered by the malate valve since MDH provides NAD+ for GDC, and thus export of malate would alleviate pressure on the respiratory chain. I do not think that stating "puts high demand on the respiratory chain" is still the way of seeing this redox echange.

Response: The text was changed to 'The conversion of Gly to Ser is a major source of NADH in leaf mitochondria in the light that is used by the respiratory chain (Krömer, 1995)…'

• 457-485: This entire paragraph does not make much sense to me. The authors performed a metabolomic analysis on seedlings at the end of the night, and they discussed a typical gly/ser ratio under photosynthetic conditions i.e. under light and therefore high photorespiratory flux. Such flux does not exist at the end of the night... What likely happens in these plants, is that there is a lack of energy to support the carbon assimilation, hence the plants accumulate amino acids as a safe source for nitrogen storage. The malate/fumarate imbalance depicted at the TCA cycle is probably incorrect. It is more likely reflecting the accumulation of photoassimilates during the day, as cytosolic/vacuolar fumarate in the WT, as compared to the mutant that is limited for redox exchange and would therefore have a lower level of fumarate throughout day and night.

Response: these are valid points and we have modified this text to focus on the specific case of leaf metabolism in the dark and how changes in non-photorespiratory serine synthesis pathways might be involved in metabolic alterations at night.

• In line with the comments above, Figures 4, 5, 6 and 7 are a bit superficial, and would require additional work to be convincing, and provide more depth to the reported study.

Response: Additional methodological details have been provided for the analyses shown in Figure 4, and the reviewer appears to have been unaware that much of the underlying data for the figure are provided in Tables S3 and S4. This is now specified in the legend for Figure 4. We are not sure why the reviewer thinks the analyses in Figures 5-7 are 'superficial', as no specific criticisms are given.

Reviewer #2 (Comments for the Author):

The authors should also pay attention to a few points below:

1) It must be clearly defined (and perhaps justified) the plant age of each analysis. Since growth parameters were obtained in 4-week-old plants it is rather strange that other analyses were performed in plants with different ages (meaning, different developmental stages) without clear justification. My feeling is that coupled with day length may explain some of the changes observed (see lines 203-231).

Response: See response to Reviewer #1 above. All experiments (apart from the second respiration experiment) were performed under long day conditions.

2) Lines 321-325: Although the authors attempted to explain why they used root tissues to analyse adenylate pools, I think it is rather complicated to connect this only measured parameter with all other data obtained in leaf tissues. I strongly recommend the authors to measure adenylate pools in leaf tissues.

Response: Adenylate pools were first measured in green seedlings, but only one repeat was analysed as we did not see any obvious difference between genotypes in these samples. If grouped in control plants (Col-0 and native RPF2) and "*atp-1* mutants" (RPF2-*atp1*-9 and RPF2-*atp1*-16) the ATP/ADP ratio average is 6.5 in the controls and ~6.6 in the "*atp1* mutants"). This data has now been added to the manuscript (Figure S10). This unexpected similarity raised the question of whether it could be due to an over-production of ATP by the chloroplast ATP synthase to compensate for an eventual ATP deficit in the "*atp1* mutants". To limit the input of the chloroplast ATP synthase, the adenylate pools were thus measured in root tissue but did not reveal any difference between genotypes either.

3) Line 225: What the authors are trying to state with "To minimize developmental stage differences between genotypes, short day conditions (8/16 hours, day/night) were applied"? This is the first time that day length is mentioned and it makes the reader wonder what are the reasons behind it.

Response: As development is slower and phenotypes are not as marked under short day conditions, the leaf sizes are more homogenous between genotypes, which is important in this experiment. This has been specified in lines 228-229.

4) Result for the RNAseq data lines 233-294. Because huge changes in transcriptomes were detected, the authors extensively described such changes in transcriptomes. However, such descriptions of the changes in transcriptomes are speculative and don't reinforce the authors' main conclusions. For the key genes discussed in the transcriptome section, please confirm the expression by other experiments to validate functionally, rather than simply discussing RNAseq data only.

Response: There are no key genes as such (we do not cite any in particular), we describe global effects and this is why RNA-seq is the best approach for this.

5) It is also important to remember that metabolite profiling here obtained by LCMS gives steady-state values and changes in those must be interpreted with caution. An increased steady-state level is usually interpreted as a block in the metabolic pathway immediately after the intermediate, in other words as reduced consumption of that intermediate. However, it could also the caused by an increased synthesis of that intermediate or by both.

Response: We agree that this is true for all metabolite level analyses and interpretation, this caveat has been noted in the text.

6) I also wonder whether the development phenotype was maternally related. Additional

experiments or text must be added to confirm that only maternal tissue contributed to the atp1 phenotype. However, the authors have only taken a snapshot picture of the seed development phenotype, and I also wonder whether this phenotype is observed since the flower appearance as well as with the number of seeds per silique.

Response: There is no reason to think the developmental phenotype is maternally inherited, as we did not modify the mitochondrial genome in these plants.

7) What is the seed germination phenotype in atp1 transformants? Does it only apply in a maternal fashion? If there is a causative link between developmental phenotype and germination rate then there would need to be a maternal link to maintain the pattern.

Response: The phenotypes associated with *RPF2-atp1* follow Mendelian inheritance; the *RPF2-atp1* transgene is dominant, as would be expected. We have not checked the seed germination phenotype of wild-type seed from *RPF2-atp1* heterozygotes, if that is the reviewer's question.

8) Throughout the main text: is development in the atp1 mutants compromised/reduced or just delayed? This could be important for trying to understand the nature of the phenotype.

Response: The development in the *atp1* mutants is dependent on the level of expression of the RPF2-*atp1* protein. For the lines described here, germination is mostly only delayed by a few days, but seed production is diminished in the RPF2-*atp1*-9 and RPF2-*atp1*-16 lines.

9) The discussion is a bit frustrating and circular mainly because it is lengthy and suffers from over-interpretation. There are some points in the discussion that have been previously commented on in the introduction. I suggest avoiding the duplicity of information.

Response: The repetitions have been removed from the discussion.

10) Because of the many indirect pieces of evidence, it is sometimes not easy to follow the idea or the conclusion. I guess that particularly non-experts in this field might have problems following the interpretation of the results. It might help to present the more direct shreds of evidence in the form of a schematic model summarizing the main results and connecting it with the phenotype observed.

Response: We feel an extra figure summarizing the results would just add more bulk to an already big manuscript

Minor comments

1) The authors are somehow mixing reducing seed yield (Supplemental Figures 3 and 4) and reduced fertility (line 245). The authors have not determined seed germination or pollen viability so they should keep the focus on seed yield.

Response: We agree, the text has been modified with this in mind.

2) The discussion is circular in places and frustrating, particularly at the beginning where there is a clear repetition of results and methods already described. Thus, the discussion should be reduced in length to give a short overview of the results and emphasize the important integrative concepts and explain how this work informs future thinking.

Response: The discussion has been shortened where possible.

3) Minor yet rather important point: It is rather strange and disappointing to see that several seminal papers/reviews from the groups around the German groups (Braun, Binder, Fernie, Maurino, Schwarzlander) working with plant mitochondria proteome and metabolism (including amino acids and organic acids) have been totally forgotten in this manuscript. I am sure the authors are aware of such work.

Response: Many papers from these groups were already cited in the first version of the manuscript (Binder, Fernie, Schwartzländer) but more citations from these groups have been added.

Reviewer #3 (Comments for the Author):

1.The mutants show increased respiration rate. Which explanation could be provided? Which pathway (cytochrome or alternative) is responsible for the increased rate? Adding inhibitors specific to the alternative pathway (SHAM) or the cytochrome pathway (KCN) could be useful, especially considering the increased abundance of AOX.

Response: given higher AOX levels in the mutants, it is probable we would measure a higher AOX activity as well, but we are not sure what these additional experiments would really add to an already substantial manuscript.

2. The involvement of the phb3 and phb3 anac017 double mutants should be better introduced in the RNA seq results. Comparison of RNA seq data from other mutants such as those affected in respiratory complexes could also be useful.

Response: We described prohibitin (PHB3) in the results section to explain why it may be involved. Additional comparisons with RNA-seq from other respiratory mutants would be interesting to do in the future but we feel that at this stage the proteomics data showing that the other respiratory complexes do not significantly respond to the reduction of ATP synthase are sufficient in this manuscript.

3. The fact that the ATP, ADP and AMP levels are close to the WT in the mutants is quite intriguing and the analyses (metabolomics, proteomics) performed do not allow to understand this point. ATP production in the chloroplasts could be increased which would explain the relatively good growth of the mutants. Why not measure the ATP content also in the leaves? Photosynthetic measurements may also help, considering the crosstalk between chloroplasts and mitochondria. This point could at least be discussed.

Response: our initial adenylate measurements in seedlings have been added to the results (see above). We feel that photosynthetic measurements are out of scope of the topic of this manuscript.

4. Discussion line 392-393: are there examples where the biogenesis of one respiratory complex is dependent on the synthesis of others?

Response: We are not aware of any such examples.

14-Nov-2023

Dr. Catherine Colas des Francs-Small
The University of Western Australia
ARC Centre of Excellence Plant Energy Biology
Perth
Australia

RE: Knockdown of mitochondrial atp1 mRNA by a custom-designed pentatricopeptide repeat protein alters F1Fo ATP synthase

Dear Dr. Colas des Francs-Small:

We are pleased to accept your manuscript for publication in the Plant Physiology. This acceptance is contingent on revision based on the comments of our reviewers.
Please attend to the minor comments by one of the reviewers and then submit final version.

Please highlight all changes and include a detailed annotation to changes to the text, with line numbers, and noting your responses to the comments.

To submit your revised manuscript, click:

Link Not Available

If you cannot return the revised manuscript within 8 weeks of receipt, please let us know. Otherwise, we will assume that you have elected not to revise the manuscript and are withdrawing it.

Thank you for allowing us to review your work. We look forward to hearing from you soon.

Sincerely,

Plant Physiology BOE

-- Reviewer comments:
Reviewer #1 (Comments for the Author):

PP2023-RA-01300D

The manuscript from Yang et al is a resubmission. The authors have provided a significantly improved manuscript, which has addressed many questions raised by the reviewers. The data are accessible, the supp. data have been polished so one can follow what it corresponds to, etc. Both text and figures have also been improved. The whole is coherent and of value for the scientific community.

A quick remark: Is there a precise thought behind the addition of "glyoxylate in the scheme figure 7? If ones add glyoxylate, many other metabolites could be added. Also, many of these reactions work both ways, hence gly-ser interconversion is not unique. I understand the authors present data supporting an adjustment of the gly-ser metabolism, but other interconversions could also be stated. I would recommend keeping it simple and having only one arrow from serine to glycine (and remove glyoxylate).

Reviewer #2 (Comments for the Author):

This is a highly updated revision of an earlier submitted manuscript which I have also worked on as a reviewer.

The authors have clearly improved their manuscript although a few points and arguments made I do not fully agree with.

Nevertheless, they expertly addressed the main concerns raised by this and the other reviewer, and their points and arguments -even though I do not entirely agree with them- are logical and more importantly intelligently presented.

The job of any reviewer is not to have the authors " do it my own way" or " to impose my way of thinking about these important

points", but to make sure that what the authors state is logical and scientifically founded.

This is clearly the big point with this resubmission and I congratulate the authors on their efforts.

Responses to the reviewers, manuscript PP2022-RA-01591 :

Response: We thank the reviewers for their thorough initial reviews and advice, and are glad they appreciated the changes made to this resubmission.

Reviewer #1 (Comments for the Author):

PP2023-RA-01300D

The manuscript from Yang et al is a resubmission. The authors have provided a significantly improved manuscript, which has addressed many questions raised by the reviewers. The data are accessible, the supp. data have been polished so one can follow what it corresponds to, etc. Both text and figures have also been improved. The whole is coherent and of value for the scientific community.

A quick remark: Is there a precise thought behind the addition of "glyoxylate in the scheme figure 7? If ones add glyoxylate, many other metabolites could be added. Also, many of these reactions work both ways, hence gly-ser interconversion is not unique. I understand the authors present data supporting an adjustment of the gly-ser metabolism, but other interconversions could also be stated. I would recommend keeping it simple and having only one arrow from serine to glycine (and remove glyoxylate).

Response: the modifications required (remove glyoxylate and remove the arrow from glycine to serine) have been made.

Reviewer #2 (Comments for the Author):

This is a highly updated revision of an earlier submitted manuscript which I have also worked on as a reviewer.

The authors have clearly improved their manuscript although a few points and arguments made I do not fully agree with.

Nevertheless, they expertly addressed the main concerns raised by this and the other reviewer, and their points and arguments -even though I do not entirely agree with them- are logical and more importantly intelligently presented.

The job of any reviewer is not to have the authors " do it my own way" or " to impose my way of thinking about these important points", but to make sure that what the authors state is logical and scientifically founded.

This is clearly the big point with this resubmission and I congratulate the authors on their efforts.

30-Nov-2023
Dr. Catherine Colas des Francs-Small
The University of Western Australia
ARC Centre of Excellence Plant Energy Biology
Perth
Australia

MSID: PP2023-RA-01300DR1
MS TITLE: Knockdown of mitochondrial atp1 mRNA by a custom-designed pentatricopeptide repeat protein alters F1Fo ATP synthase

Dear Dr. Colas des Francs-Small:

I am pleased to inform you that your revised manuscript "Knockdown of mitochondrial atp1 mRNA by a custom-designed pentatricopeptide repeat protein alters F1Fo ATP synthase" has been accepted for publication in Plant Physiology. Your manuscript will be checked for consistency with journal formatting and image standards by one of our Science Editors, who will contact you shortly. They may have a number of queries and suggestions for you. Please keep in mind that they work to increase the online visibility of your article and its attraction for readers from the widest of backgrounds. You will have the opportunity to review any revisions, and I hope you will work constructively with them to get the most out of your research. You will also have the opportunity to review all changes when you receive the author proofs for your article.

Once validated for publication, your article will be sent to a Science Editor for a final review of your manuscript and source files. After your final files are uploaded, your article will appear in the next available issue and the online Preview version of your article will be posted after your signed Copyright and Checklist forms and final source files are received.

Please be sure to include the following in your manuscript file:
Harvard style citations
List of all author contributions and funding information at the beginning of manuscript after the titles and author list.
List all supplemental materials associated with the article, with titles, at the end of the ms, before the references and acknowledgments section.
Complete Figure Legends for all figures appearing in the article listed before the Literature Cited.

Thank you and congratulations on your Plant Physiology paper!

Sincerely,
Plant Physiology Board of Editors

=====
FIRST AUTHOR PROFILES
=====

We recognize the hard work that goes into being First Author of a Plant Physiology paper by publishing a "First Author Feature" for each First Author alongside your paper.

If the first author(s) would like to submit information for a feature on Facebook and Twitter, fill out the form by clicking on the link below:

Plant Physiology First Author Feature Form: https://forms.office.com/r/ZBCGZfhm8F
If you cannot access the form through the link above, please contact Rachel at rbelsky@aspb.org.

To upload an image of yourself to accompany your profile, please put your photo in the correct journal folder at the link below. You must label your image: Author First Name Last Name Article Title.

First Author Profile Image Dropbox: https://www.dropbox.com/scl/fo/xz029p7qjr2jml2rp2kmq/h?dl=0&rlkey=6fkxcaqmxx35ap152b2osy1fz

=====
IMPORTANT REMINDER: PEER REVIEW REPORTS
=====

If you opted to publish a peer review report along with your article during the original submission process, it will be prepared by the editorial staff and publicly posted with your manuscript, inside the zip file that contains any other supplemental material. As a reminder, the peer review report is a public record of all comments from editors and reviewers, as well as your prior responses, as you received them in the decision letters for each draft of your manuscript. If you agreed to publish this report and have changed your mind, or are not sure if you selected this option, please contact the editorial office as soon as possible before signing the license agreement from our publisher.

-- Reviewer comments:
